# Functional outcomes and quality of life following open versus laparoscopic versus robot-assisted versus transanal total mesorectal excision in rectal cancer patients: a systematic review and meta-analysis

**DOI:** 10.1007/s00464-024-10934-4

**Published:** 2024-06-19

**Authors:** Ritch T. J. Geitenbeek, Thijs A. Burghgraef, Carmen A. Moes, Roel Hompes, Adelita V. Ranchor, Esther C. J. Consten, the MIRECA study group, G. J. D. van Acker, T. S. Aukema, H. J. Belgers, F. H. Beverdam, J. G. Bloemen, K. Bosscha, S. O. Breukink, P. P. L. O. Coene, R. M. P. H. Crolla, P. van Duijvendijk, E. B. van Duyn, I. F. Faneyte, S. A. F. Fransen, A. A. W. van Geloven, M. F. Gerhards, W. M. U. van Grevenstein, K. Havenga, I. H. J. T. de Hingh, C. Hoff, G. Kats, J. W. A. Leijtens, M. F. Lutke Holzik, J. Melenhorst, M. M. Poelman, A. Pronk, A. H. W. Schiphorst, J. M. J. Schreinemakers, C. Sietses, A. B. Smits, I. Somers, E. J. Spillenaar-Bilgen, H. B. A. C. Stockmann, A. K. Talsma, P. J. Tanis, J. Tuynman, E. G. G. Verdaasdonk, F. A. R. M. Warmerdam, H. L. van Westreenen, D. D. E. Zimmerman

**Affiliations:** 1 Department of Surgery, Meander Medical Center, Maatweg 3, 3813 TZ Amersfoort, The Netherlands; 2 Department of Surgery, University of Groningen, University Medical Center Groningen, Hanzeplein 1, 9713 GZ Groningen, The Netherlands; 3 Department of Surgery, University of Amsterdam, University Medical Center Amsterdam, Amsterdam, The Netherlands; 4 Department of Surgery, Amsterdam Cancer Center, Amsterdam, The Netherlands; 5 Department of Health Psychology, University of Groningen, University Medical Center Groningen, Groningen, the Netherlands

**Keywords:** Total mesorectal excision, Rectal cancer, Systematic review, Meta-analysis, Functional outcomes, Quality of life

## Abstract

**Background:**

The standard surgical treatment for rectal cancer is total mesorectal excision (TME), which may negatively affect patients’ functional outcomes and quality of life (QoL). However, it is unclear how different TME techniques may impact patients’ functional outcomes and QoL. This systematic review and meta-analysis evaluated functional outcomes of urinary, sexual, and fecal functioning as well as QoL after open, laparoscopic (L-TME), robot-assisted (R-TME), and transanal total mesorectal excision (TaTME).

**Methods:**

A systematic review and meta-analysis, based on the preferred reporting items for systematic reviews and meta-analysis statement, were conducted (PROSPERO: CRD42021240851). A literature review was performed (sources: PubMed, Medline, Embase, Scopus, Web of Science, and Cochrane Library databases; end-of-search date: September 1, 2023), and a quality assessment was performed using the Methodological index for non-randomized studies. A random-effects model was used to pool the data for the meta-analyses.

**Results:**

Nineteen studies were included, reporting on 2495 patients (88 open, 1171 L-TME, 995 R-TME, and 241 TaTME). Quantitative analyses comparing L-TME vs. R-TME showed no significant differences regarding urinary and sexual functioning, except for urinary function at three months post-surgery, which favoured R-TME (SMD [CI] –0 .15 [− 0.24 to − 0.06], *p* = 0.02; *n* = 401). Qualitative analyses identified most studies did not find significant differences in urinary, sexual, and fecal functioning and QoL between different techniques.

**Conclusions:**

This systematic review and meta-analysis highlight a significant gap in the literature concerning the evaluation of functional outcomes and QoL after TME for rectal cancer treatment. This study emphasizes the need for high-quality, randomized-controlled, and prospective cohort studies evaluating these outcomes. Based on the limited available evidence, this systematic review and meta-analysis suggests no significant differences in patients' urinary, sexual, and fecal functioning and their QoL across various TME techniques.

**Supplementary Information:**

The online version contains supplementary material available at 10.1007/s00464-024-10934-4.

Total mesorectal excision (TME) is the standard surgical treatment of rectal cancer, which can be performed using open, laparoscopic (L-TME), robot-assisted (R-TME), and transanal TME (TaTME) [1]. All three minimally invasive techniques are considered standard care and offer comparable intraoperative, postoperative, or oncological outcomes when performed by experienced surgeons [2, ]. With comparable oncological outcomes, patients often prioritize postoperative recovery and quality of life (QoL) in their decision making. As survival rates improve, patients may have to cope with reduced functional outcomes and lower QoL for an extended period [3, ]. Therefore, it is essential to assess the evidence regarding functional outcomes and QoL of these techniques.

Research has mostly focused on the oncological results of minimally invasive techniques for rectal cancer treatment, leaving limited evidence concerning their functional outcomes and QoL [4]. The available evidence is contradictory, with some studies suggesting that R-TME and TaTME techniques increase the nerve-sparing dissection rate and improve functional outcomes by optimizing the visualization of adjacent structures and enhancing precision in dissection around the distal rectum [5, 6]. However, other studies have found no evidence for the advantages of R-TME [4, 7] and some have even suggested unfavourable outcomes of TaTME due to anal canal dilatation and the risk of nerve injury during the learning curve [8, 9]. The existing systematic reviews [7, 10–13] have not included the most recently published studies and only compare two minimally invasive techniques. Furthermore, these reviews also rely on retrospectively gathered data about functional outcomes and QoL.

To address this gap, an updated, comprehensive systematic review and meta-analysis of prospectively collected data were conducted to examine the functional outcomes and QoL of open, L-TME, R-TME, and TaTME techniques for rectal cancer treatment.

## Methods

This systematic review and meta-analysis were conducted following Preferred Reporting Items for Systematic Reviews and Meta-Analysis (PRISMA) guidelines and in line with the protocol agreed by all authors. The study protocol was written a priori following the PRISMA-P statement and registered in the International prospective register of systematic reviews (Prospero #240,851) [14].

### Data selection

In accordance with the population, intervention, comparison, outcomes, and study design (PICOS) framework, the study eligibility criteria were selected. *Population*: adult patients with rectal cancer undergoing TME. Interventions: open TME, L-TME, R-TME, and TaTME. *Comparisons*: studies were deemed eligible independently of head-to-head comparisons. *Outcomes*: functional outcomes, measured by any validated scale, defined as the concrete consequences of TME surgery in rectal cancer patients. These include urinary, sexual, and fecal function. QoL, measured by any validated scale, is defined as the personal perception of the impact of illness or treatments on physical, psychological, and social well-being [15]. The various domains of QoL were analyzed separately. These include physical, social, and psychological QoL. However, due to variations in questionnaires used per study and definitions used, functional outcomes and QoL were reported as described in individual studies, and different reporting outcomes were evaluated for inclusion on a case-by-case basis. *Study design*: randomized-controlled trials (RCTs), prospective studies, and retrospective studies reporting from a prospective database. Excluded were studies that met at least one of the following criteria: (a) not published in English, French, German, or Spanish, (b) less than six months follow-up, (c) case reports, case series, letters, editorials, conference abstracts, commentaries, and reviews, and (d) full-text unavailable. In case of overlapping populations between studies, the study with the largest sample size and longest follow-up were included.

The systematic search was supported by a librarian experienced in assisting systematic reviews and peer-reviewed by a second independent librarian. The search dates were from January 1st, 2000, to September 1st, 2023. The pre-defined complete search strategy prioritized sensitivity above specificity and is available in Supplementary File 1. Studies were assessed for eligibility through searches of the PubMed, Medline, Embase, Scopus, Web of Science, and Cochrane Library databases. In addition, databases of ongoing (unpublished) trials (i.e., World Health Organisation (WHO) Registry Network (including ClinicalTrials.gov) and PROSPERO were searched. Two independent reviewers (RG, CM) identified potentially eligible studies through title and abstract screening using Rayyan QCRI, a web-based software management program for systematic reviews. Disagreements were solved through discussion, in which two additional authors were involved (AR and EC). For literature saturation, reference lists of included studies were hand-searched for additional relevant studies using systematic “snowball” procedure guidelines [16].

### Data extraction

Two independent reviewers (RG, CM) extracted data from the included studies using a standardized data extraction form. Prior to data extraction, training was provided for using the form. The tabulated data were used for evidence synthesis and quality assessment. Disagreements were solved through discussion, in which two additional authors were involved (AR and EC). For each eligible study, the following information was collected: study characteristics (first author, journal and year of publication, country, study design, aim, primary/secondary outcomes, in/exclusion criteria, study period, type of surgery, number of patients, length of follow-up), patient demographics (gender, age, body mass index, tumour location), preoperative data (disease stage, neoadjuvant treatment, American Society of Anaesthesiologists (ASA) score), intraoperative data (number of surgeons, surgeon experience, operation time, type of procedure, stoma rate, conversion rate), postoperative data (circumferential resection margin involvement and Clavien-Dindo classification of postoperative complications), and functional outcomes and QoL. In case of missing relevant data, the study's corresponding authors were requested for additional data. A reminder email was sent up to three times.

### Quality assessment

The quality of included studies was independently appraised by two reviewers (RG, CM) using a Modified Methodological Items for Non-Randomized Studies (MINORS) tool [17] (Supplementary File 2 and 3). The adjusted MINORS tool consists of 12 questions for comparative studies (scale: 0-1-2, max score: 24). Studies with scores ≤ 18 were considered of low quality. In the MINORS tool item assessing whether the follow-up period was appropriate to the aim of the study, the cut-off was a priori set at 12 months postoperatively. This follow-up period was chosen as most events influencing morbidity or mortality will likely occur within one year postoperatively. Criteria 9–12 of the MINORS tool were assessed in the case of comparative studies. The control and study group were considered contemporary if these were managed no more than five years apart. Disagreements were resolved through discussion, in which two additional authors were involved (AR and EC). The Grading of Recommendations Assessment, Development and Evaluation working group approach (GRADE) was used to assess the quality of evidence for the functional outcomes and QoL [18]. The quality of evidence was reported as high, moderate, low, or very low.

### Data analysis

A meta-analysis for functional outcomes was performed if ≥ 3 comparative studies reported functional outcomes of urinary, sexual, or fecal function. A meta-analysis for QoL was performed if ≥ 3 comparative studies reported QoL data based on validated questionnaires such as the European Organization for Research and Treatment of Cancer (EORTC) questionnaires. Data from studies was only included in the meta-analyses if the number of patients that participated in the questionnaires was available. The Cochrane Handbook 6 was used as a guideline for these analyses [19]. Standardized mean differences with 95% confidence intervals (CIs) were calculated for continuous variables. As we anticipated considerable between-study heterogeneity, a random-effects model was used to pool effect sizes and checked using a fixed-effect model. Given its robust performance in continuous outcome data, the restricted maximum likelihood estimator was used to calculate the heterogeneity variance τ^2^ [20]. Knapp-Hartung adjustments were used to calculate the confidence interval around the pooled effect [21]. If the requested data were unavailable, mean(s.d.) values were calculated for the overall analysis, if possible [22]. Outcome reporting bias was assessed by comparing outcomes reported in study protocols to the final published article. As subgroup analyses depend on the statistical power, no subgroup analyses were performed since the number of studies is small (*k* =  < 10). Following Cochrane guidelines, we did not investigate publication bias as our search considered less than ten studies for each data comparison.

A qualitative synthesis of functional outcomes and QoL was performed to present the findings of studies that were excluded from the meta-analysis, following the European Social Research Council Guidance on the Conduct of Narrative Synthesis in Systematic Reviews [23]. Categorical data were summarized using numbers and percentages. Continuous variables were summarized as means and standard deviations (SDs) or median and interquartile ranges (IQRs). Functional outcomes and QoL were compared between techniques. Studies comparing techniques head-to-head were prioritized.

## Results

### Study selection

**Fig. 1:**
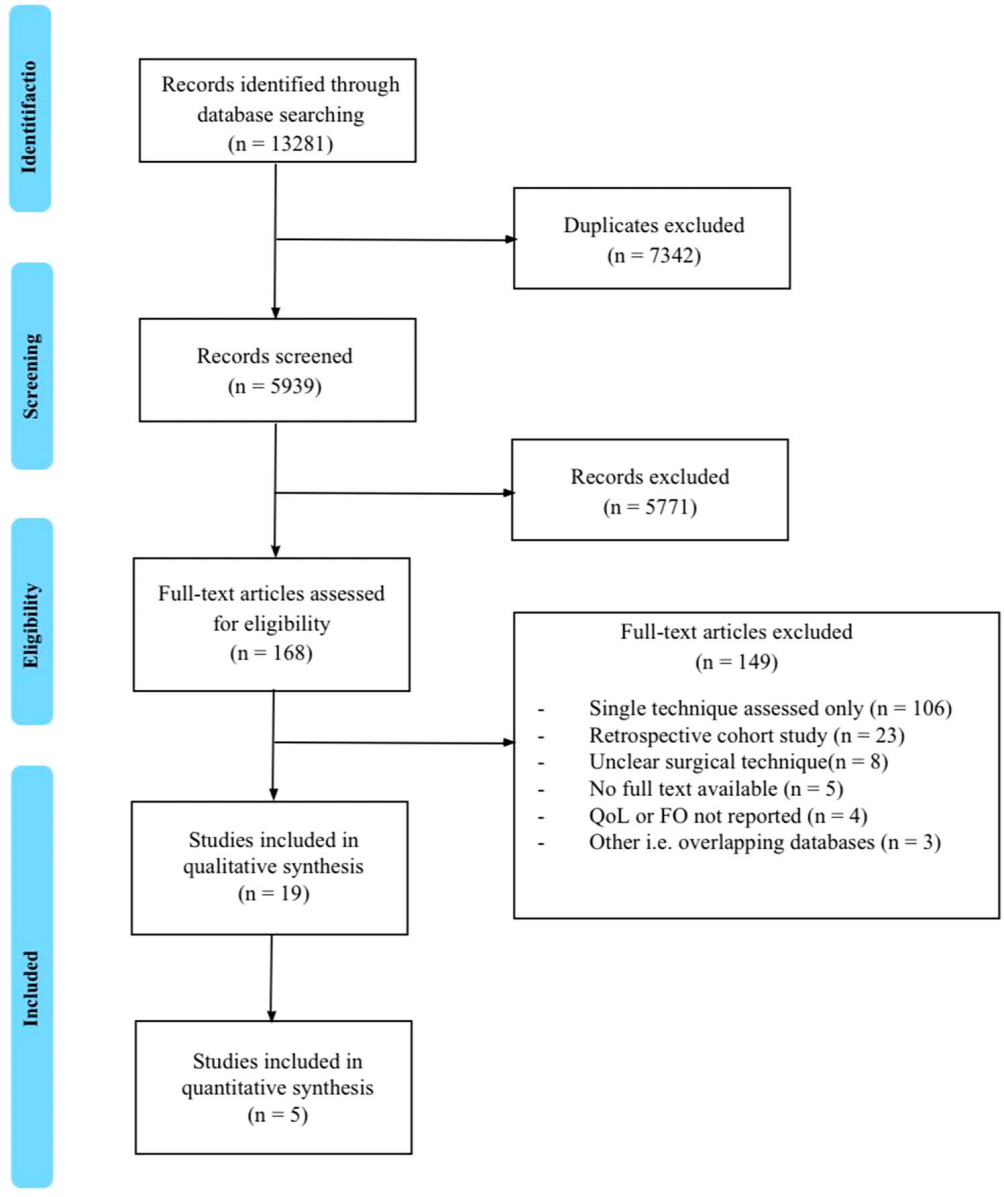
Flowchart depicting study selection. *FO* functional outcomes; *n* number; *QoL *quality of life

After the removal of duplicates, a total of 5939 citations were identified (Fig. 1). Following the abstract and full-text screening, 19 studies were included for qualitative analyses [2, 24–41] and five studies for quantitative analyses [24, 33, 35, 40, 41]. Demographics of the included studies are provided in Table 1. Functional outcomes and QoL were reported as primary outcomes in over 70% of the included studies (14/19). Patient demographics and preoperative characteristics are provided in Supplementary Table 1. Operative data and postoperative outcomes are provided in Supplementary Table 2.

**Table 1:** Demographics of the studies included in this review

First author	Year	Country	Study design	Outcome	Study period	Follow-up	# total	# O-TME	# L-TME	# R-TME	# TaTME
D’Annibale et al.	2013	Italy	NRRS	Functional	L-TME: 2004–2012 R-TME 2006–2012	12 months	100	NA	50	50	NA
Feng et al.	2022	China	RCT	Functional	12/2013–12/2016	12 months	347	NA	173	174	NA
Galata et al.	2019	Germany	NRPS	Functional	02/2016–12/2017	12 months	51	NA	33	18	NA
Grass et al.	2021	Germany	NRPS	Functional	01/2014–02/2018	12 months	120	NA	NA	55	65
Heijden et al.	2021	Netherlands	NRPS	Functional/QoL	2014–2019	12 months	110	NA	55	NA	55
Hur et al.	2013	Korea	NRPS	Functional	04/2009–08/2010	12 months	97	41	56	NA	NA
Jayne et al.	2017	UK	RCT	Functional	01/2011–09/2014	6 months	471	NA	234	237	NA
Kim et al. (AoS)	2018	Korea	RCT	QoL	02/2012–03/2015	12 months	139	NA	73	66	NA
Kim et al. (ASO)	2012	Korea	NRPS	Functional	06/2009–11/2009	12 months	69	NA	39	30	NA
Kim et al. (CRD)	2018	Korea	NRPS	Functional/QoL	2009–2013	12 months	260	NA	130	130	NA
Liu et al.	2022	China	NRPS	Functional	01/2017–01/2020	12 months	135	NA	45	90	NA
Macháčková et al.	2022	Czech Republic	NRRS	Functional	03/2016–06/2018	24 months	66	NA	39	27	NA
Mei et al.	2021	China	NRPS	QoL	01/2015–12/2019	12 months	180	NA	90	NA	90
Miura et al.	2022	Japan	NRPS	Functional	08/2016–04/2020	6 months	52	NA	11	33	8
Ng et al.	2013	China	NRPS	QoL	07/2006–07/2008	12 months	74	25	49	NA	NA
Ozeki et al.	2016	Japan	NRPS	Functional	09/2011–01/2013	12 months	37	22	NA	15	NA
Park et al.	2014	Korea	NRRS	Functional	02/2009–12/2010	12 months	64	NA	32	32	NA
Rubinkiewicz et al.	2019	Poland	NRPS	Functional	2013–2017	6 months	46	NA	23	NA	23
Tang et al.	2022	China	NRPS	Functional	06/2018–07/2020	12 months	77	NA	39	38	NA
Total.							2495	88	1171	995	241

*L-TME* Laparoscopic total mesorectal excision; *NA* not applicable; *NRPS* non-randomized prospective study; *NRRS* non-randomized retrospective study reporting from prospective database; *O-TME* open total mesorectal excision; *QoL* quality of life; *R-TME* robotic total mesorectal excision; *RCT* randomized-controlled trial; *TaTME* transanal total mesorectal excision; *#* number

### Functional outcomes

Functional outcomes, including urinary, sexual, and fecal function, were assessed in 16 studies [2, 24–30, 32–35, 37, 39–41], which incorporated the results of the treatment of 2102 patients (Table 2). These studies included 63 open TME, 959 L-TME, 929 R-TME, and 151 TaTME patients.

**Table 2:** Overview of functional outcomes of the included studies

Urinary function		IPSS					
Study	Technique	Post-op (timepoint not specified)	1 m	3 m	6 m	12 m	24 m
Annibale et al.	L-TME/R-TME		NS			NS	
Feng et al.	L-TME/R-TME		RS	RS	RS		
Galata et al.	L-TME/R-TME					∆ NS	
Grass et al.	R-TME/TaTME					∆ ♂ TaS, ∆ ♀ NS	
Jayne et al.*	L-TME/R-TME				NS*		
Kim et al. (ASO)	L-TME/R-TME		NS	RS	NS	NS	
Kim et al. (CRD)	L-TME/R-TME			NS	RS	NS	
Liu et al.	L-TME/Microhand/da Vinci			RS^3^, RS^4^			
Macháčková et al.	L-TME/R-TME				NS	NS	NS
Miura et al.	L-TME/R-TME/TaTME	NS^1^, NS^2^					
Park et al.	L-TME/R-TME			NS	NS	NS	
Tang et al.	L-TME/R-TME			NS	RS	RS	

Sexual function		IIEF					FSFI			
Study	Technique	1 m	3 m	6 m	12 m	24 m	3 m	6 m	12 m	24 m
Feng et al.	L-TME/R-TME		RS	RS	RS		RS	RS	RS	
Galata et al.	L-TME/R-TME				NS				∆ NS	
Grass et al.	R-TME/TaTME								∆ NS	
Jayne et al.*	L-TME/R-TME			NS*				NS*		
Kim et al. (ASO)	L-TME/R-TME	NS	NS	NS	NS					
Kim et al. (CRD)	L-TME/R-TME		NS	NS	NS					
Liu et al.	L-TME/Microhand/da Vinci			RS^1^, RS^2^				NS^1^, NS^2^		
Macháčková et al.	L-TME/R-TME			NS	NS	NS		NS	NS	NS
Park et al.	L-TME/R-TME		NS	RS	NS					
Tang et al.	L-TME/R-TME		NS	RS	NS					

Fecal function		LARS		Wexner	
Study	Technique	6 m postoperatively or after stoma closure	12 m	6 m postoperatively or after stoma closure	12 m
Grass et al.	R-TME/TaTME		∆ RS		∆ NS
Heijden et al.	L-TME/TaTME		LS		
Miura et al.	L-TME/R-TME/TaTME	NS^1^, NS^2^		NS^1^, NS^2^	
Rubinkiewicz et al.*	L-TME/TaTME	NS*		NS*	

*IPSS* International Prostate Symptom Score; *L-TME *laparoscopic total mesorectal excision; *m* months; *NS* non-significant; *Post-op* postoperative; *R-TME* robotic total mesorectal excision; *RS* robot significantly better; *TaTME* transanal total mesorectal excision; *TaS* transanal significantly better; *FSFI* female sexual function index; *IIEF* International Index of Erectile Function; *LS* laparoscopic significantly better; *LARS* low anterior resection syndrome

*Calculated L-TME minus R-TME; ♂, male; ♀, female; ^1^L-TME vs. R-TME; ^2^R-TME vs. TaTME; ^3^Microhand vs. L-TME; ^4^da Vinci vs. L-TME; ∆*,* delta. The reported significance of the questionnaire outcomes was reported as described by the reviewed studies and according to comparison to preoperative baseline questionnaire outcomes, unless otherwise specified

*Calculated L-TME minus R-TME; ^1^Microhand vs. L-TME; ^2^da Vinci vs. L-TME; ∆*,* delta

*Provided median LARS/Wexner scores; ∆*,* delta; ^1^L-TME vs. R-TME; ^2^R-TME vs. TaTME

#### Urinary function

**Fig. 2:**
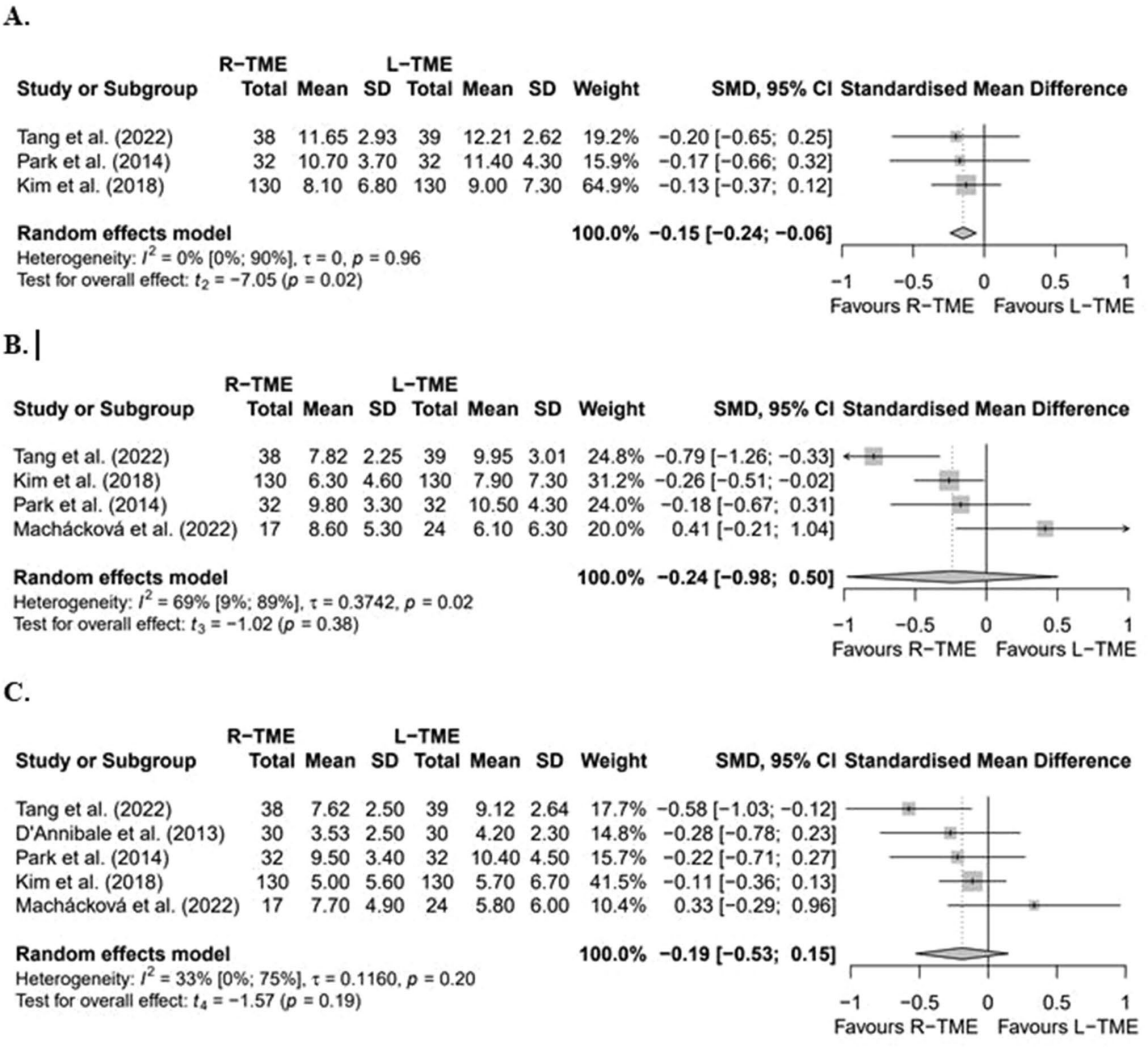
Forest plots of standardized mean differences of IPSS scores of the included studies at three months (**A**), six months (**B**) and 12 months (**C**). *L-TME* laparoscopic total mesorectal excision; *R-*TME robotic total mesorectal excision; *SD* standard deviation;* SMD* standardised mean difference

To assess urinary function, the international prostate symptom scores (IPSS) questionnaire was used by a total of 14 studies [24–27, 29, 30, 32–35, 37, 39–41] (12 non-RCTs, 2 RCTs, 1946 patients) (Supplementary Table 3). Five studies [24, 33, 35, 40, 41] comparing L-TME vs. R-TME provided sufficient data to perform a meta-analysis (Fig. 2). The meta-analysis showed that R-TME had significantly better IPSS at three months postoperatively [33, 40, 41] (standardized mean difference (SMD − 0.15 [95% CI − 0.24; − 0.06], *p* = 0.02; *n* = 401). There were no significant differences at six months [33, 35, 40, 41] (SMD − 0.24 [95% CI − 0.98; 0.50], *p* = 0.38; *n* = 442) or 12 months postoperatively [24, 33, 35, 40, 41] (SMD − 0.19 [95% CI − 0.53; 0.15], *p* = 0.19; *n* = 502). Heterogeneity was absent at three months (*I*^2^ = 0%), substantial at six months (*I*^2^ = 69%), and moderate at 12 months (*I*^2^ = 33%). Findings of the qualitative synthesis of studies excluded from meta-analyses were in line with the results of the meta-analyses, with all three studies [25, 32, 34] that reported IPSS at three months postoperatively also revealing significantly better outcomes of R-TME compared to L-TME. Regarding TaTME, Miura et al. [37] found no significant differences at six months postoperatively between TaTME and L-TME or R-TME. However, Grass et al. [27] found significantly better IPSS outcomes in males at 12 months after TaTME compared to R-TME.

#### Sexual function

**Fig. 3:**
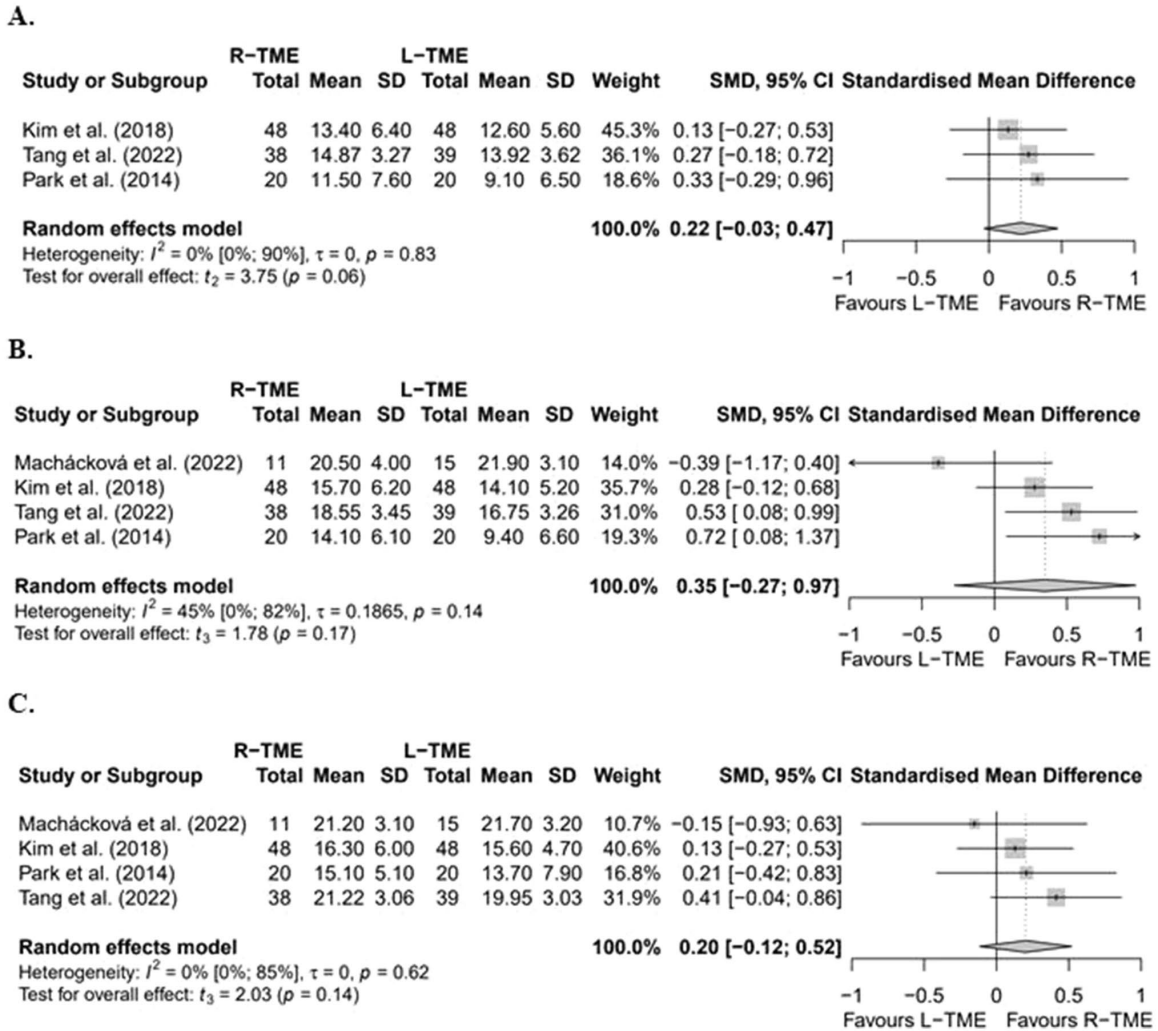
Forest plots of standardized mean differences of IIEF scores of the included studies at three months (**A**), six months (**B**) and 12 months (**C**). *L-TME* laparoscopic total mesorectal excision; *R-TME *robotic total mesorectal excision; *SD* standard deviation;* SMD* standardised mean difference

To assess male sexual function, the International Index of Erectile Function scores (IIEF) questionnaire was used by a total of 12 studies [25–27, 29, 30, 32–35, 39–41] (10 non-RCTs, 2 RCTs, 1794 patients) Supplementary Table 4. Four studies [33, 35, 40, 41] comparing L-TME vs. R-TME provided sufficient data to perform a meta-analysis (Fig. 3). The meta-analysis showed no differences at three months [33, 40, 41] (SMD 0.22, [95% CI − 0.03 to 0.47], *p* = 0.06; *n* = 213), six months [33, 35, 40, 41] (SMD 0.35 [95% CI − 0.27; 0.97], *p* = 0.17; *n* = 239) or 12 months postoperatively [33, 35, 40, 41] (SMD 0.20 [95% CI − 0.12; 0.52], *p* = 0.14; *n* = 239). Heterogeneity was absent at three months (*I*^2^ = 0%), moderate at six months (*I*^2^ = 45%), and absent at 12 months (*I*^2^ = 0%). In addition, six studies [25–27, 30, 34, 35] assessed female sexual functioning with the female sexual function index scores (FSFI) questionnaire (4 non-RCTs, 2 RCTs, 1190 patients). Of these studies, five [25, 26, 30, 34, 35] compared L-TME to R-TME, of which four [26, 30, 34, 35] reported no differences between techniques. Feng et al. [25] reported significantly better FSFI at three, six, and 12 months postoperatively after R-TME compared to L-TME. Regarding TaTME, Grass et al. [27] reported no significant differences between TaTME and R-TME at 12 months postoperatively.

#### Fecal function

To assess fecal function, the Low Anterior Resection Syndrome score (LARS) questionnaire was used by four studies [2, 27, 28, 37] (all non-RCTs, 328 patients) (Supplementary Table 5). Two of these studies, when analysing outcomes 12 months after surgery, demonstrated significantly worse results for TaTME in comparison to L-TME [28] and R-TME [27], respectively. Nonetheless, it is important to note that in both of these studies, the sample size was relatively limited, with only approximately 50 patients in each group. Furthermore, it's worth noting that in the study by Grass et al. [27], the TaTME group received neoadjuvant radiotherapy twice as frequently as the R-TME group. This disparity in neoadjuvant radiotherapy may potentially impact sphincter function. Conversely, the remaining two studies [2, 37] failed to identify any notable distinctions between TaTME and L-TME or R-TME. Additionally, three studies [2, 27, 37] assessed fecal function using the Wexner score questionnaire (all non-RCTs, 218 patients), reporting no significant differences between techniques.

### QoL outcomes

QoL outcomes were assessed in five studies [28, 31, 33, 36, 38], which incorporated treatment results of 763 patients (Table 3). These studies included 25 open TME, 397 L-TME, 196 R-TME, and 145 TaTME patients.

**Table 3:** Overview of quality of life of the included studies

Study	Technique	Global health score			
		4 m	6 m	8 m	12 m
Heijden et al	L-TME/TaTME				NS
Kim et al. (AoS)	L-TME/R-TME				NS
Kim et al. (CRD)	L-TME/R-TME				NS
Mei et al	L-TME/TaTME		NS		TaS
Ng et al	O-TME/L-TME	LS		LS	NS

*L-TME* Laparoscopic total mesorectal excision; *LS* laparoscopic significantly better; *m* months; *NR* not reported; *NS* non-significant; *O-TME* open total mesorectal excision; *Post-op* postoperative; *Pre-op* preoperative; *R-TME* robotic total mesorectal excision; *TaTM,* transanal total mesorectal excision; *TaS* transanal significantly better

#### Overall quality of life

To assess overall QoL, the Global Health scores of the EORTC Core Quality of Life questionnaire (QLQ-C30, QLQ-CR29, and QLQ-CR38) were used by five studies [28, 31, 33, 36, 38] (4 non-RCTs, 1 RCT, 763 patients) Supplementary Table 6. Of these studies, one [38] reported significantly better outcomes of L-TME vs. open TME, and one [36] reported significantly better Global Health scores at 12 months postoperatively in TaTME patients compared to L-TME patients. A list of subscores reported for questionnaires used in the included studies is provided in Supplementary Table 7. In the majority of included studies, when comparing the QLQ-C30, QLQ-CR29, and QLQ-CR38 across distinct domains (such as physical, emotional, social, role, and cognitive) and symptoms, no significant differences were reported.

#### Quality of evidence

As only three of the included studies were RCTs, all were assessed with the MINORS tool to ensure comparability. The included studies were of heterogeneous quality (Supplementary File 3). Consequently, the certainty in evidence according to GRADE regarding urinary and sexual function was rated as very low. The mean MINORS tool score was 17.8 ± 2.1, indicating a low methodological quality of the included studies, and only four studies were considered high-quality (score of ≥ 20). Reasons for the low score were mainly unblinded assessment of the outcomes of interest, loss to follow-up > 5%, and failure to report a prospective calculation of the study size.

## Discussion

This systematic review and meta-analysis summarizes the prospective evidence regarding functional outcomes and QoL after open, L-TME, R-TME, and TaTME. Overall, analyses indicated no statistically significant differences in patients' urinary, sexual, and anorectal function and QoL between these techniques. Although there may be a slight advantage in the recovery of urinary function for R-TME patients three months postoperatively, the evidence quality was graded as very low. Consequently, we emphasize the need for future high-quality, prospective trials with adequate statistical power to provide a more definitive assessment of functional outcomes and QoL.

In terms of urinary function, quantitative analyses suggest a potential early advantage for R-TME, as indicated by the faster recovery of IPSS scores at three months postoperatively compared to L-TME. However, this advantage did not persist at six and 12 months postoperatively. These findings align with the qualitative analyses of studies excluded from the meta-analyses. However, contrasting outcomes were reported in previous systematic reviews and meta-analyses [42–45], emphasizing better results of R-TME and TaTME at six and 12 months postoperatively compared to L-TME. These findings may be attributed to technical variances between the techniques, with novel TME approaches potentially offering superior visualization and a reduced risk of neurovascular injuries leading to urinary incontinence and sexual dysfunction [46, 47]. However, the current study found no evidence suggesting urinary function favoring the novel TME approaches. Several considerations merit attention when interpreting these findings. Most studies in this review consistently found no significant differences in urinary function between the various techniques. This lack of differences may be attributed to the multifaceted nature of urinary dysfunction, making it challenging to isolate the surgical technique's impact on this outcome. Urinary function is inherently subjective and strongly influenced by patient-related factors such as expectations and coping skills. It is conceivable that surgical technique may exert only limited influence on postoperative urinary function. Moreover, potential design and statistical issues may have also contributed to some studies reporting no significant differences. A subset of studies was at risk of bias due to a lack of adjustment for confounding factors, such as significantly higher rates of neoadjuvant treatment in the TaTME groups and elevated baseline IPSS scores in the R-TME groups, both recognized as confounders for postoperative anorectal and urogenital outcomes [7, 48–50]. Additionally, in most included studies, urinary dysfunction was not a primary endpoint, rendering them underpowered to detect statistically significant differences. Notably, in contrast to the other studies included in the meta-analysis, the study by Machakova et al. [35] reported urinary outcomes favouring L-TME instead of R-TME. Due to the limited number of studies included in the meta-analysis, this small sample study may have significantly influenced the outcomes of the meta-analysis regarding urinary and sexual function.

Regarding sexual function, quantitative analyses did not reveal any significant differences between the techniques. These results concur with the qualitative analyses of studies excluded from the meta-analyses. However, contrasting evidence from prior systematic reviews and meta-analyses suggested significantly better R-TME outcomes than L-TME at three and six months postoperatively [42–45]. Despite this, most studies in our review found no significant differences in sexual function among the techniques. This could be attributed to low response rates and the use of different questionnaires for men and women, leading to small sample sizes and underpowered studies. Nonetheless, certain studies did report better sexual function after R-TME and TaTME compared to conventional laparoscopic surgery. As discussed previously, if there even are any differences favoring novel TME techniques, this may be because of technical differences between the techniques, with R-TME and TaTME potentially reducing the occurrence of autonomic nerve injury [51]. Discrepancies in sexual outcomes may also be due to variations in anastomosis rates between the techniques. R-TME and TaTME could facilitate the construction of a low anastomosis in patients who would otherwise require an abdominoperineal resection (APR) [52]. APR includes extralevator perineal excision, associated with a higher risk of neurovascular bundle injuries and worse sexual outcomes. Patients undergoing APR also receive a permanent stoma, negatively affecting sexual function [53]. Notably, some studies observed improved sexual function after R-TME and TaTME exclusively in male patients, hinting at potential advantages in specific patient subgroups, such as those with a narrow pelvic region.

In terms of fecal function, qualitative analyses failed to uncover any significant differences among the techniques. Nonetheless, certain studies reported significant differences in fecal function after R-TME and TaTME compared to conventional laparoscopic surgery. Van der Heijden et al. included 55 patients receiving TaTME and L-TME and reported worse LARS scores for TaTME at 12 months postoperatively. This contradicts prior studies suggesting worse anorectal outcomes for TaTME only during the initial six months of recovery, attributed to anal stretch and dilatation [54, 55]. The differences in fecal function outcomes after R-TME and TaTME could potentially be attributed to patient selection bias. Surgeons may have chosen R-TME or TaTME for patients with a very low tumour height, avoiding intersphincteric APR. Consequently, these groups may consist of more patients receiving a low anastomosis, which, due to reduced rectal reservoir capacity, could lead to major LARS [56].

Regarding QoL, the qualitative analyses did not discern any differences between the techniques. Nevertheless, some studies did report significant differences in QoL between TME techniques. For instance, Ng et al. compared 25 patients receiving open surgery with 49 patients receiving L-TME and found significantly better outcomes for L-TME at four and eight months postoperatively. These findings were postulated to reflect the superior short-term clinical outcomes of minimally invasive surgery versus open surgery [57–61]. Additionally, Mei et al. reported improved outcomes for TaTME compared to laparoscopic surgery at 12 months postoperatively, although this study juxtaposed TaTME with L-APR. However, as previously mentioned, differences between type of procedure performed could have a notable impact on patients’ QoL. The absence of significant differences in QoL might be due to the inherent complexity of this subjective outcome. Compared to urinary and sexual function, QoL is influenced by even more multifaceted factors, including coping abilities and social environment, and the technique of surgery performed may only have a small effect on global QoL.

The absence of significant differences unearthed by this review could be due to the poor quality of available evidence. Most of the literature consisted of small non-randomized trials that assessed functional outcomes or QoL as secondary endpoints, resulting in a substantial lack of power, particularly regarding open and TaTME. Acknowledging the limitations in the number of patients receiving open and transanal TME in the studies included in this review, this study aimed to provide an overview of the currently available evidence regarding all TME techniques and focused exclusively on studies with prospective data collection for functional outcomes or QoL. Incorporating studies with retrospective data collection could have increased the overall number of patients in our analysis, thereby enhancing statistical power. However, retrospective data collection methods are susceptible to recall bias and may not provide a comprehensive and accurate representation of the patients' true QoL and functional outcomes. Thus, prioritizing prospective data collection enhances the reliability and validity of our findings. Nevertheless, to comprehensively assess functional outcomes and QoL, future studies should be meticulously designed, prospective, and sufficiently powered. Moreover, patient-related factors such as coping skills, social context, and psychological well-being largely influence subjective functional outcomes and QoL, highlighting the necessity of considering these confounders when evaluating these parameters. Beyond patient-related factors, the choice of surgical procedure and clinical outcomes, including postoperative morbidity, can significantly impact reported outcomes, making it difficult to isolate the exclusive effect of surgical technique. Notably, whilst the presence of a temporary or permanent ostomy may significantly affect patients’ functional outcomes and QoL, the studies included in this review often did not report the numbers of patients receiving an ostomy, nor were these factors considered when evaluating patient reported outcomes. Thus, future studies are strongly recommended to account for these confounding factors when assessing functional outcomes and QoL. Additionally, several studies in our analyses compared experienced laparoscopic surgeons to surgeons’ initial experience with robot-assisted surgery, necessitating adjustment for the learning curve's effects [26, 30]. A fair evaluation of techniques should involve comparing surgeons with similar experience levels. Consequently, we eagerly await the forthcoming results of the Vantage trial [62], which will provide prospective insights into the functional and QoL outcomes of surgeons who have surpassed the learning curve. Lastly, functional outcomes and QoL assessments often lacked clear definitions and employed questionnaires not validated for rectal cancer patients, resulting in considerable heterogeneity between groups and rendering meaningful statistical comparisons difficult.

## Conclusion

In summary, this systematic review and meta-analysis revealed a significant gap in the literature concerning the evaluation of functional outcomes and QoL after TME for rectal cancer treatment. Existing studies are limited in number and frequently reported functional outcomes and QoL as secondary endpoints. Acknowledging the limited strength of the evidence, this systematic review and meta-analysis found no significant differences in urinary, sexual, and anorectal function, as well as QoL, across various TME techniques. While innovative techniques like R-TME and TaTME have shown oncological safety, it is imperative to undertake high-quality, prospective trials with sufficient statistical power to assess functional outcomes and QoL comprehensively. Such studies should also account for the impact of the learning curve and baseline patient characteristics, ensuring a more robust evaluation of these critical parameters in rectal cancer surgery.

### Supplementary Information

Below is the link to the electronic supplementary material.

Supplementary file1 (DOCX 121 KB)

## Data Availability

Data used in this systematic review will be made available upon reasonable request. The pre-defined complete search strategy is available as a Supplementary File.
