# Supplementary material for: Functional outcomes and quality of life following open versus laparoscopic versus robot-assisted versus transanal total mesorectal excision in rectal cancer patients: a systematic review and meta-analysis
Source: Surg Endosc. 2024 Jun 19;38(8):4431–44. doi: 10.1007/s00464-024-10934-4 (PMC11289076; doi:10.1007/s00464-024-10934-4)
Supplement: Supplementary file 1 — Supplementary file1 (DOCX 121 KB) [file 464_2024_10934_MOESM1_ESM.docx]

**Supplementary File**: PRISMA Checklist

| **Section/topic** | **#** | **Checklist item** | **Reported on page #** |
| --- | --- | --- | --- |
| **TITLE** | | |  |
| Title | 1 | Identify the report as a systematic review, meta-analysis, or both. | 1 |
| **ABSTRACT** | | |  |
| Structured summary | 2 | Provide a structured summary including, as applicable: background; objectives; data sources; study eligibility criteria, participants, and interventions; study appraisal and synthesis methods; results; limitations; conclusions and implications of key findings; systematic review registration number. | 4-5 |
| **INTRODUCTION** | | |  |
| Rationale | 3 | Describe the rationale for the review in the context of what is already known. | 6-7 |
| Objectives | 4 | Provide an explicit statement of questions being addressed with reference to participants, interventions, comparisons, outcomes, and study design (PICOS). | 6-7 |
| **METHODS** | | |  |
| Protocol and registration | 5 | Indicate if a review protocol exists, if and where it can be accessed (e.g., Web address), and, if available, provide registration information including registration number. | 8 |
| Eligibility criteria | 6 | Specify study characteristics (e.g., PICOS, length of follow-up) and report characteristics (e.g., years considered, language, publication status) used as criteria for eligibility, giving rationale. | 8-9 |
| Information sources | 7 | Describe all information sources (e.g., databases with dates of coverage, contact with study authors to identify additional studies) in the search and date last searched. | 9-10 |
| Search | 8 | Present full electronic search strategy for at least one database, including any limits used, such that it could be repeated. | Suppl. File 1 |
| Study selection | 9 | State the process for selecting studies (i.e., screening, eligibility, included in systematic review, and, if applicable, included in the meta-analysis). | 8-12 |
| Data collection process | 10 | Describe method of data extraction from reports (e.g., piloted forms, independently, in duplicate) and any processes for obtaining and confirming data from investigators. | 9-10 |
| Data items | 11 | List and define all variables for which data were sought (e.g., PICOS, funding sources) and any assumptions and simplifications made. | 9-10 |
| Risk of bias in individual studies | 12 | Describe methods used for assessing risk of bias of individual studies (including specification of whether this was done at the study or outcome level), and how this information is to be used in any data synthesis. | 10-11 |
| Summary measures | 13 | State the principal summary measures (e.g., risk ratio, difference in means). | 11-12 |
| Synthesis of results | 14 | Describe the methods of handling data and combining results of studies, if done, including measures of consistency (e.g., I^2^) for each meta-analysis. | 11-12 |

| **Section/topic** | **#** | **Checklist item** | **Reported on page #** |
| --- | --- | --- | --- |
| Risk of bias across studies | 15 | Specify any assessment of risk of bias that may affect the cumulative evidence (e.g., publication bias, selective reporting within studies). | 11 |
| Additional analyses | 16 | Describe methods of additional analyses (e.g., sensitivity or subgroup analyses, meta-regression), if done, indicating which were pre-specified. | 11 |
| **RESULTS** | | |  |
| Study selection | 17 | Give numbers of studies screened, assessed for eligibility, and included in the review, with reasons for exclusions at each stage, ideally with a flow diagram. | 13, Figure 1 |
| Study characteristics | 18 | For each study, present characteristics for which data were extracted (e.g., study size, PICOS, follow-up period) and provide the citations. | 13, Table 1 |
| Risk of bias within studies | 19 | Present data on risk of bias of each study and, if available, any outcome level assessment (see item 12). | 16, Suppl. File 3 |
| Results of individual studies | 20 | For all outcomes considered (benefits or harms), present, for each study: (a) simple summary data for each intervention group (b) effect estimates and confidence intervals, ideally with a forest plot. | 13-16, Table 2-3, Suppl. Table 3-6 |
| Synthesis of results | 21 | Present results of each meta-analysis done, including confidence intervals and measures of consistency. | 13-16, Figure 2-3 |
| Risk of bias across studies | 22 | Present results of any assessment of risk of bias across studies (see Item 15). | 16, Suppl. File 3 |
| Additional analysis | 23 | Give results of additional analyses, if done (e.g., sensitivity or subgroup analyses, meta-regression [see Item 16]). | NA |
| **DISCUSSION** | | |  |
| Summary of evidence | 24 | Summarize the main findings including the strength of evidence for each main outcome; consider their relevance to key groups (e.g., healthcare providers, users, and policy makers). | 17-21 |
| Limitations | 25 | Discuss limitations at study and outcome level (e.g., risk of bias), and at review-level (e.g., incomplete retrieval of identified research, reporting bias). | 17-21 |
| Conclusions | 26 | Provide a general interpretation of the results in the context of other evidence, and implications for future research. | 21 |
| **FUNDING** | | |  |
| Funding | 27 | Describe sources of funding for the systematic review and other support (e.g., supply of data); role of funders for the systematic review. | 2 |

*From:*  Moher D, Liberati A, Tetzlaff J, Altman DG, The PRISMA Group (2009). Preferred Reporting Items for Systematic Reviews and Meta-Analyses: The PRISMA Statement. PLoS Med 6(7): e1000097. doi:10.1371/journal.pmed1000097

For more information, visit: **www.prisma-statement.org**.

**Supplementary File 1:** Search syntax

**PubMed**

("Rectal Neoplasms"[Mesh] OR ((“rectal”[tiab] OR “rectum”[tiab] OR “mesorectal”[tiab] OR “recti”[tiab]) AND (“neoplasm*”[tiab] OR “cancer*”[tiab] OR “tumor*”[tiab] OR “tumour*”[tiab] OR “malignan*” [tiab] OR “carcinoma*” [tiab] OR “oncolog*” [tiab] OR “mass*”[tiab])))

AND

(((“total mesorect*”[tiab] OR “total meso-rect*”[tiab]) AND (“excision*”[tiab] OR “removal*”[tiab] OR “surger*”[tiab] OR “resection*”[tiab] OR “extirpation”[tiab])) OR “TME”[tiab])

AND

("Proctectomy"[Mesh] OR “proctectom*”[tiab] OR “rectum resection”[tiab] OR "Laparoscopy"[Mesh] OR “laparoscop*”[tiab] OR “laparoscopic TME”[tiab] OR “Laparoscopic Total Mesorectal Excision*”[tiab] OR “celioscop*”[tiab] OR “coelioscop*”[tiab] OR “abdominoscop*” [tiab] OR

“peritoneoscop*” [tiab] OR "Robotic Surgical Procedures"[Mesh] OR “robotic-assisted surger*”[tiab] OR “robot-assisted surger*”[tiab] OR “da vinci”[tiab] OR “robot surger*”[tiab] OR “robotic surgical procedure*”[tiab] OR “robot surgical procedure*”[tiab] OR “robot enhanced surger*”[tiab] OR

“robotic surger*”[tiab] OR “robot-aided surger*”[tiab] OR “robotic-aided surger*”[tiab] OR “robotically-assisted surger*”[tiab] OR "Transanal Endoscopic Surgery"[Mesh] OR “transanal endoscopic surger*”[tiab] OR “TME”[tiab] OR “TaTME”[tiab] OR

“transanal total mesorectal excision*”[tiab] OR “transanal TME”[tiab] OR “transanal minimally invasive surger*”[tiab] OR “tamis”[tiab] OR “minimally invasive procedure*”[tiab] OR “minimal access surger*”[tiab] OR "minimally invasive surger*"[tiab] OR “minimally invasive method*”[tiab] OR “minimally invasive technique*”[tiab] OR "Laparotomy"[Mesh] OR “laparotom*”[tiab] OR “open surger*”[tiab])

**Embase**

('rectum tumor'/exp OR ((‘rectal’:ti,ab OR ‘rectum’:ti,ab OR ‘mesorectal’:ti,ab OR ‘recti’:ti,ab) AND (‘neoplasm*’:ti,ab OR ‘cancer*’:ti,ab OR ‘tumor*’:ti,ab OR ‘tumour*’:ti,ab OR ‘malignan*’:ti,ab OR ‘carcinoma*’:ti,ab OR ‘oncolog*’:ti,ab OR ‘mass*’:ti,ab)))

AND

('total mesorectal excision'/exp OR ((‘total mesorect*’:ti,ab OR ‘total meso-rect*’:ti,ab) AND (‘excision*’:ti,ab OR ‘removal*’:ti,ab OR ‘surger*’:ti,ab OR ‘resection*’:ti,ab OR ‘extirpation’:ti,ab)) OR ‘TME’:ti,ab)

AND

('rectum resection'/exp OR ‘proctectom*’:ti,ab OR 'laparoscopy'/exp OR 'laparoscopic total mesorectal excision'/exp OR ‘laparoscop*’:ti,ab OR ‘laparoscopic TME’:ti,ab OR ‘laparoscopic total mesorectal excision’:ti,ab OR ‘coelioscop*’:ti,ab OR ‘celioscop*’:ti,ab OR ‘abdominoscop*’:ti,ab OR ‘peritoneoscop*’:ti,ab OR ‘robot assisted surgery'/exp OR ‘robotic surger*’:ti,ab OR ‘robot enhanced surger*’:ti,ab OR ‘robotic surgical procedure*’:ti,ab OR ‘robot surgical procedure*’:ti,ab OR ‘robotic-assisted surger*’:ti,ab OR ‘da vinci’:ti,ab OR ‘robot surger*’:ti,ab OR ‘robotic surger*’:ti,ab OR ‘robot-assisted surger*’:ti,ab OR ‘robot-aided surger*’:ti,ab OR ‘robotic-aided surger*’:ti,ab OR ‘robotically-assisted surger*’:ti,ab OR 'transanal total mesorectal excision'/exp OR 'transanal endoscopic surgery'/exp OR 'transanal minimally invasive surgery'/exp OR 'minimally invasive procedure'/exp OR ‘minimal access surgery'/exp OR ‘transanal endoscopic surger*’:ti,ab OR ‘TaTME’:ti,ab OR ‘transanal TME’:ti,ab OR ‘transanal total mesorectal excision*’:ti,ab OR ‘transanal minimally invasive surger*’:ti,ab OR ‘tamis’:ti,ab OR ‘minimally invasive procedure*’:ti,ab OR ‘minimally invasive surger*’:ti,ab OR ‘minimal access surger*’:ti,ab OR ‘minimally invasive method*’:ti,ab OR ‘minimally invasive technique*’:ti,ab OR 'laparotomy'/exp OR 'open surgery'/exp OR ‘laparotom*’:ti,ab OR ‘open surger*’:ti,ab)

**Web of Science**

TS=((“rectal” OR “rectum” OR “mesorectal” OR “recti”) AND (“neoplasm*” OR “cancer*” OR “tumor*” OR “tumour*” OR “malignan*” OR “carcinoma*” OR “oncolog*” OR “mass*”))

AND

TS=(((“total mesorect*” OR “total meso-rect*”) AND (“excision*” OR “removal*” OR “surger*” OR “resection*” OR “exstirpation”)) OR “TME”)

AND

TS=(“proctectom*” OR “rectum resection” OR “laparoscop*” OR “laparoscopic TME” OR “Laparoscopic Total Mesorectal Excision*” OR “celioscop*” OR “coelioscop*” OR “abdominoscop*” OR “peritoneoscop*” OR “robotic-assisted surger*” OR “robot-assisted surger*” OR “da vinci” OR “robot surger*” OR “robotic surgical procedure*” OR “robot surgical procedure*” OR “robot enhanced surger*” OR “robotic surger*” OR “robot-aided surger*” OR “robotic-aided surger*” OR “robotically-assisted surger*” OR “transanal endoscopic surger*” OR “TME” OR “TaTME” OR “transanal total mesorectal excision*” OR “transanal TME” OR “transanal minimally invasive surger*” OR “tamis” OR “minimally invasive procedure*” OR “minimal access surger*” OR "minimally invasive surger*" OR “minimally invasive method*” OR “minimally invasive technique*” OR “laparotom*” OR “open surger*”)

**Cochrane library**

ID Search

#1 MeSH descriptor: [Rectal Neoplasms] explode all trees

#2 (rectal):ti,ab,kw

#3 (rectum):ti,ab,kw

#4 (mesorectal):ti,ab,kw

#5 (recti):ti,ab,kw

#6 (neoplasm*):ti,ab,kw

#7 (cancer*):ti,ab,kw

#8 (tumour*):ti,ab,kw

#9 (malignan*):ti,ab,kw

#10 (carcinoma*):ti,ab,kw

#11 (oncolog*):ti,ab,kw

#12 (mass*):ti,ab,kw

#13 (#1 OR ((#2 OR #3 OR #4 OR #5) AND (#6 OR #7 OR #8 OR #9 OR #10 OR #11 OR #12)))

#14 (total mesorect*):ti,ab,kw

#15 (total meso-rect*):ti,ab,kw

#16 (excision*):ti,ab,kw

#17 (removal*):ti,ab,kw

#18 (surger*):ti,ab,kw

#19 (resection*):ti,ab,kw

#20 (exstirpation*):ti,ab,kw

#21 (TME):ti,ab,kw

#22 (((#14 OR #15) AND (#16 OR #17 OR #18 OR #19 OR #20)) OR #21)

#23 MeSH descriptor: [Proctectomy] explode all trees

#24 (proctectom*):ti,ab,kw

#25 (rectum resection):ti,ab,kw

#26 MeSH descriptor: [Laparoscopy] explode all trees

#27 (laparoscop*):ti,ab,kw

#28 (laparoscopic TME):ti,ab,kw

#29 (laparoscopic total mesorectal excision*):ti,ab,kw

#30 (celioscop*):ti,ab,kw

#31 (coelioscop*):ti,ab,kw

#32 (abdominoscop*):ti,ab,kw

#33 (peritoneoscop*):ti,ab,kw

#34 MeSH descriptor: [Robotic Surgical Procedures] explode all trees

#35 (robotic-assisted surger*):ti,ab,kw

#36 (robot-assisted surger*):ti,ab,kw

#37 (da vinci):ti,ab,kw

#38 (robot surger*):ti,ab,kw

#39 (robotic surgical procedure*):ti,ab,kw

#40 (robot surgical procedure*):ti,ab,kw

#41 (robot enhanced surger*):ti,ab,kw

#42 (robotic surger*):ti,ab,kw

#43 (robot-aided surger*):ti,ab,kw

#44 (robotically-assisted surger*):ti,ab,kw

#45 MeSH descriptor: [Transanal Endoscopic Surgery] explode all trees

#46 (transanal endoscopic surger*):ti,ab,kw

#47 (TME):ti,ab,kw

#48 (TaTME):ti,ab,kw

#49 (transanal total mesorectal excision*):ti,ab,kw

#50 (transanal TME):ti,ab,kw

#51 (transanal minimally invasive surger*):ti,ab,kw

#52 (tamis):ti,ab,kw

#53 (minimally invasive procedure*):ti,ab,kw

#54 (minimal access surger*):ti,ab,kw

#55 (minimally invasive method*):ti,ab,kw

#56 (minimally invasive technique*):ti,ab,kw

#57 MeSH descriptor: [Laparotomy] explode all trees

#58 (laparotom*):ti,ab,kw

#59 (open surger*):ti,ab,kw

#60 (#23 OR #24 OR #25 OR #26 OR #27 OR #28 OR #29 OR #30 OR #31 OR #32 OR #33 OR #34 OR #35 OR #36 OR #37 OR #38 OR #39 OR #40 OR #41 OR #42 OR #43 OR #44 OR #45 OR #46 OR #47 OR #48 OR #49 OR #50 OR #51 OR #52 OR #53 OR #54 OR #55 OR #56 OR #57 OR #58 OR #59)

#61 (#13 AND #22 AND #60)

**Filters and limits**

Limits were imposed for date of publication. This review included studies published between January 1st, 2000, to September 1st, 2023 in the search strategy.

| **Supplementary File 2:** Modified Methodological Items for Non-Randomized Studies (MINORS) tool | | | |
| --- | --- | --- | --- |
| **Items** | **Sub-category questions** | **Explanation** | **Score** |
| 1 | **A clearly stated aim**: the question addressed should be precise and relevant in the light of available literature. | Clear description of relevant patient characteristics, interventions, control groups and outcomes provided.  One or more of the above were unclear or not reported. | 2      0 |
| 2 | **Inclusion of consecutive patients**: all patients potentially fit for inclusion (satisfying the criteria for inclusion) have been included in the study during the study period (no exclusion or details about the reasons for exclusion). | Inclusion of consecutive patients, details and numbers of excluded patients provided.  Inclusion of consecutive patients, details and numbers of excluded patients not provided.  No description provided or no inclusion of consecutive patients. | 2        1    0 |
| 3 | **Prospective collection of data**: data were collected according to a protocol established before the beginning of the study. | Prospective study design with protocol established before the beginning of the study.  Prospective study design without protocol established before the beginning of the study.  Retrospective analysis of prospectively collected data with or without protocol established before the beginning of the study. | 2  1  0 |
| 4 | **Endpoints appropriate to the aim of the study**: unambiguous explanation of the criteria used to evaluate the main outcome which should be in accordance with the question addressed by the study. Also, the endpoints should be assessed on an  intention-to-treat basis. | Unambiguous explanation of the criteria used to evaluate the quality of life and/or functional outcomes (i.e. for low anterior resection syndrome analysis using the low anterior resection syndrome questionnaire, providing definitions of major low anterior resection syndrome and scoring).  Ambiguous explanation of the criteria used to evaluate the quality of life and/or functional outcomes.  No description provided. | 2            1  0 |
| 5 | **Unbiased assessment of the study endpoint**: blind evaluation of objective endpoints and double-blind evaluation of subjective endpoints. Otherwise the reasons for not blinding should be stated. | Blind evaluation of quality of life and/or functional outcomes of questionnaires or telephone interviews.  Evaluation of quality of life and/or functional outcomes of questionnaires or telephone interviews performed not blinded, with reasons for not blinding provided.  Unclear if evaluation of quality of life and/or functional outcomes questionnaires or telephone interviews was blinded. | 2  1  0 |
| 6 | **Follow-up period appropriate to the aim of the study**: the follow-up should be sufficiently long to allow the assessment of the main endpoint and possible adverse events. | Follow-up of ≥ 12 months for quality of life and/or functional outcomes.  Follow-up of < 12 months for quality of life and/or functional outcomes.  No description provided. | 2  1  0 |
| 7 | **Loss to follow up less than 5%**: all patients should be included in the follow up. Otherwise, the proportion lost to follow up should not exceed the proportion experiencing the major endpoint. | Loss to follow up reported and ≤ 5%.  Loss to follow up reported and > 5%.  No description provided. | 2  1  0 |
| 8 | **Prospective calculation of the study size**: information of the size of detectable difference of interest with a calculation of 95% confidence interval, according to the expected incidence of the outcome event, and information about the level for statistical significance and estimates of power when comparing the outcomes. | Prospective calculation of the study size provided with information of the size of detectable difference of interest of quality of life and/or functional outcomes with a calculation of 95% confidence interval, according to the expected incidence of the outcome event, and information about the level for statistical significance and estimates of power when comparing the outcomes.  Prospective calculation of the study size provided with information of the size of detectable difference of interest of other outcomes with a calculation of 95% confidence interval, according to the expected incidence of the outcome event, and information about the level for statistical significance and estimates of power when comparing the outcomes.  No description provided. | 2  1  0 |
| *Additional criteria in the case of comparative study* | | | |
| 9 | **An adequate control group**: having a gold standard diagnostic test or therapeutic intervention recognized as the optimal intervention according to the available published data. | Control group consisting of open, laparoscopic, robotic or transanal total mesorectal excision.  Control group consisting of other than gold standard therapeutic intervention.  No description provided. | 2  1  0 |
| 10 | **Contemporary groups**: control and studied group should be managed during the same time period (no historical comparison). | Control and studied group are contemporary, managed during the same time period.  Control and studied group are contemporary, managed with time period difference of > 5 years.  No description provided. | 2  1  0 |
| 11 | **Baseline equivalence of groups**: the groups should be similar regarding the criteria other than the studied endpoints. Absence of confounding factors that could bias the interpretation of the results. | Baseline equivalence of groups, no significant differences reported between groups for confounding factors that could bias the interpretation of the results.  Baseline equivalence of groups, significant differences reported between groups for confounding factors that could bias the interpretation of the results.  No description provided. | 2  1  0 |
| 12 | **Adequate statistical analyses**: whether the statistics were in accordance with the type of study with calculation of confidence intervals or relative risk. | Adequate statistical analyses, the statistics were in accordance with the type of study with calculation of confidence intervals or relative risk.  Inadequate statistical analyses, the statistics were not in accordance with the type of study with calculation of confidence intervals or relative risk.  No description provided. | 2  1  0 |
| Modified Methodological Items for Non-Randomized Studies (MINORS) checklist tool. Questions used per category and explanatory note are provided. The items are scored 0 (not reported), 1 (reported but inadequate) or 2 (reported and adequate). | | | |

| **Supplementary File 3:** Quality assessment of the included studies using the Modified Methodological Items for Non-Randomized Studies (MINORS) checklist | | | | | | | | | | | | | | |
| --- | --- | --- | --- | --- | --- | --- | --- | --- | --- | --- | --- | --- | --- | --- |
|  |  | Modified Methodological Items for Non-Randomized Studies (MINORS) checklist question | | | | | | | | | | | | |
| Study | 1 | 2 | 3 | 4 | 5 | 6 | 7 | 8 |  | 9 | 10 | 11 | 12 | Total |
| D'Annibale et al | 2 | 1 | 0 | 1 | 0 | 2 | 0 | 0 | Additional criteria in the case of comparative study | 2 | 2 | 1 | 2 | 13 |
| Feng et al | 2 | 2 | 2 | 2 | 2 | 1 | 2 | 1 |  | 2 | 2 | 2 | 2 | 22 |
| Galata et al | 2 | 1 | 1 | 1 | 0 | 2 | 1 | 0 |  | 2 | 2 | 2 | 2 | 16 |
| Grass et al | 2 | 2 | 2 | 2 | 0 | 2 | 2 | 0 |  | 2 | 2 | 1 | 2 | 19 |
| Heijden et al | 2 | 2 | 2 | 2 | 0 | 2 | 2 | 0 |  | 2 | 2 | 2 | 2 | 20 |
| Hur et al | 2 | 2 | 2 | 2 | 0 | 2 | 0 | 0 |  | 2 | 2 | 2 | 2 | 18 |
| Jayne et al | 2 | 2 | 2 | 2 | 1 | 1 | 2 | 1 |  | 2 | 2 | 2 | 2 | 21 |
| Kim et al. (2018) | 2 | 2 | 2 | 2 | 0 | 2 | 1 | 1 |  | 2 | 2 | 2 | 2 | 20 |
| Kim et al. (2012) | 2 | 2 | 2 | 2 | 0 | 2 | 0 | 0 |  | 2 | 2 | 2 | 2 | 18 |
| Kim et al. (2018) | 2 | 2 | 0 | 2 | 0 | 2 | 2 | 0 |  | 2 | 2 | 2 | 2 | 18 |
| Liu et al | 2 | 2 | 1 | 2 | 0 | 2 | 0 | 2 |  | 2 | 2 | 1 | 2 | 18 |
| Macháčková et al | 2 | 0 | 0 | 2 | 0 | 2 | 2 | 0 |  | 2 | 2 | 2 | 2 | 16 |
| Mei et al | 2 | 2 | 2 | 2 | 0 | 2 | 1 | 0 |  | 2 | 2 | 1 | 2 | 18 |
| Miura et al | 2 | 2 | 2 | 2 | 0 | 1 | 1 | 0 |  | 2 | 2 | 1 | 2 | 17 |
| Ng et al | 2 | 1 | 1 | 2 | 0 | 2 | 0 | 2 |  | 2 | 2 | 2 | 2 | 18 |
| Ozeki et al | 2 | 1 | 1 | 2 | 0 | 2 | 1 | 0 |  | 2 | 2 | 1 | 2 | 16 |
| Park et al | 2 | 1 | 1 | 2 | 0 | 2 | 1 | 2 |  | 2 | 2 | 2 | 2 | 19 |
| Rubinkiewicz et al | 2 | 1 | 1 | 2 | 0 | 1 | 0 | 0 |  | 2 | 2 | 2 | 2 | 15 |
| Tang et al | 2 | 1 | 1 | 2 | 0 | 2 | 1 | 0 |  | 2 | 2 | 2 | 2 | 17 |
| *NA,* not applicable  The Modified Methodological Items for Non-Randomized Studies (MINORS) checklist is a current research standard for conducting systematic reviews which are based on non-randomized studies and was used to rate study the quality of all studies included in this review. The results of the MINORS tool indicate risk of bias and applicability concerns. This study used a modified MINORS tool using categorical questions adjusted to study design of the included studies. The global ideal score being 16 for non-comparative studies and 24 for comparative studies. | | | | | | | | | | | | | | |

| **Supplementary Table 1:** Patients demographics and preoperative characteristics of included studies | | | | | | | | | | | | | | | | | | | |  |
| --- | --- | --- | --- | --- | --- | --- | --- | --- | --- | --- | --- | --- | --- | --- | --- | --- | --- | --- | --- | --- |
|  |  | Gender, n (%) | |  | |  | |  |  | | Stage, n (%) | | | | | ASA Classification, n (%) | | | | |
| First author | Technique | Male | Female | | Age, mean (years) | BMI, mean | Neoadjuvant therapy, n (%) | | | Distance to anal verge, mean cm (SD) (range) | 0 | 1 | 2 | 3 | 4 | I | II | III | IV | |
| D’Annibale et al. | L-TME | 30 (60.0) | 20 (40.0) | | 65.72 ± 11.6 |  | 28 (56.0) | | | 17 LR |  |  |  |  |  |  |  |  |  | |
|  | R-TME | 30 (60.0) | 20 (40.0) | | 66 ± 12.1 |  | 34 (68.0) | | | 33 LR |  |  |  |  |  |  |  |  |  | |
| Feng et al. | L-TME | 113 (65.3) | 60 (34.7) | | 59.5 (10.9) |  | 35 (20.2) | | | 3.6 (0.7) |  | 66 (38.2) | 57 (32.9) | 50 (28.9) |  | 116 (67.1) | 50 (28.9) | 7 (4.0) |  | |
|  | R-TME | 108 (62.1) | 66 (37.9) | | 58.2 (9.6) |  | 37 (21.3) | | | 3.5 (0.7) |  | 58 (33.3) | 56 (32.2) | 60 (34.5) |  | 124 (71.3) | 46 (26.4) | 4 (2.3) |  | |
| Galata et al. | L-TME | 21 (63.6) | 12 (36.4) | | 62.3 ± 13.7 (33–83) | 27.4 ± 5.5 (16.8–39.8) | 20 (60.6) | | | 7.7 ± 3.3 (1–15) |  | 12 (36.4) | 9 (27.3) | 9 (27.3) | 3 (9.1) |  | 21 (63.6) | 12 (36.4) |  | |
|  | R-TME | 10 (55.6) | 8 (44.4) | | 60.0 ± 11.8 (34–76) | 26.0 ± 4.0 (19.5–34.7) | 11 (61.1) | | | 8.5 ± 4.0 (2–15) |  | 5 (27.8) | 5 (27.8) | 7 (38.9) | 1 (5.6) |  | 15 (83.3) | 3 (16.7) |  | |
| Grass et al. | R-TME | 38 (69.1) | 17 (30.9) | | 59.2 ± 11.9 | 27.2 ± 5.3 | 18 (32.7) | | | 6.7 ± 5.3 |  |  |  |  |  |  |  |  |  | |
|  | TaTME | 40 (61.5) | 25 (38.5) | | 66.6 ± 10.4 | 25.4 ± 4.0 | 41 (63.1) | | | 5.5 ± 2.4 |  |  |  |  |  |  |  |  |  | |
| Heijden et al. | L-TME | 31 (56.4) | 24 (43.6) | | 62.0 (8) |  | 34 (61.8) | | | 21 (45) LR |  | 10 (18.2) | 15 (27.3) | 27 (49.1) | 3 (5.5) |  |  |  |  | |
|  | TaTME | 33 (60.0) | 22 (40.0) | | 62.1 (8) |  | 31 (56.4) | | | 21 (45) LR |  | 13 (23.6) | 12 (21.8) | 25 (45.5) | 5 (9.1) |  |  |  |  | |
| Hur et al. | O-TME | 26 (63.4) | 15 (36.6) | | 55 (28–74) | 23.6 ± 2.7 | 14 (34.1) | | | 14 (34.1) LR |  |  |  |  |  |  |  |  |  | |
|  | L-TME | 36 (64.3) | 20 (35.7) | | 58 (30–74) | 23.8 ± 2.7 | 19 (33.9) | | | 19 (33.9) LR |  |  |  |  |  |  |  |  |  | |
| Jayne et al. | L-TME | 159 (67.9) | 75 (32.1) | | 65.5 (11.93) |  | 108 (46.2) | | | 61 (26.5) LR |  |  |  |  |  | 52 (22.2) | 124 (53.0) | 52 (22.2) | 1 (0.4) | |
|  | R-TME | 161 (67.9) | 76 (32.1) | | 64.4 (10.98) |  | 111 (46.8) | | | 57 (24.2) LR |  |  |  |  |  | 39 (16.5) | 150 (63.3) | 46 (19.4) | 0 (0.0) | |
| Kim et al. (AoS) | L-TME | 52 (71.2) | 21 (28.8) | | 59.7 (11.7) | 23.6 (3.0) | 58 (79.5) | | | 35 (48.0) LR |  |  |  |  |  | 30 (41.1) | 43 (58.9) |  |  | |
|  | R-TME | 51 (77.3) | 15 (22.7) | | 60.4 (9.7) | 24.1 (3.3) | 51 (77.3) | | | 33 (50.0) LR |  |  |  |  |  | 20 (30.3) | 46 (69.7) |  |  | |
| Kim et al. (ASO) | L-TME | 20 (51.3) | 19 (48.7) | | 56.85 ± 11.14 | 24.01 ± 2.19 | 12 (30.8) | | | 8 (20.5) LR |  |  |  |  |  | 39 (100.0) | 0 (0.0) |  |  | |
|  | R-TME | 18 (60.0) | 12 (40.0) | | 54.13 ± 8.52 | 24.36 ± 2.44 | 10 (33.3) | | | 6 (20.0) LR |  |  |  |  |  | 29 (96.7) | 1 (3.3) |  |  | |
| Kim et al. (CRD) | L-TME | 95 (73.1) | 35 (26.9) | | 60.0 (9.3) | 23.3 (2.9) | 61 (46.9) | | | 6.3 (2.6) | 6 (4.6) | 45 (34.6) | 40 (30.8) | 39 (30.0) |  | 70 (53.6) | 46 (35.4) | 14 (10.8) |  | |
|  | R-TME | 95 (73.1) | 35 (26.9) | | 60.5 (10.1) | 23.7 (3.2) | 63 (48.5) | | | 5.9 (2.7) | 9 (6.9) | 45 (34.6) | 29 (22.3) | 47 (36.2) |  | 71 (54.6) | 48 (36.9) | 11 (8.5) |  | |
| Liu et al. | L-TME | 25 (55.6) | 20 (44.4) | | 58.53 ± 8.78 | 23.3 ± 2.37 | 27 (60.0) | | | 5.23 ± 0.92 |  |  |  |  |  | 27 (60.0) | 18 (40.0) | 0 (0.0) |  | |
|  | Microhand | 25 (58.1) | 18 (41.9) | | 58.81 ± 7.06 | 22.70 ± 1.66 | 24 (55.8) | | | 5.19 ± 0.64 |  |  |  |  |  | 25 (58.1) | 17 (39.5) | 1 (2.4) |  | |
|  | Da Vinci | 27 (57.4) | 20 (42.6) | | 58.7 ± 7.63 | 22.50 ± 1.67 | 26 (55.3) | | | 5.15 ± 0.83 |  |  |  |  |  | 28 (59.6) | 18 (38.3) | 1 (2.1) |  | |
| Macháčková et al. | L-TME | 24 (61.5) | 15 (38.5) | | 62.0 ± 9.1 | 26.9 ± 4.5 | 24 (61.5) | | | 7.1 ± 1.8 |  |  |  |  |  | 0 (0.0) | 24 (61.5) | 14 (35.9) | 1 (2.56) | |
|  | R-TME | 17 (62.9) | 10 (37.0) | | 61.0 ± 10.9 | 26.8 ± 4.7 | 19 (70.4) | | | 7.4 ± 3.1 |  |  |  |  |  | 0 (0.0) | 14 (51.8) | 13 (48.2) | 0 (0.0) | |
| Mei et al. | L-TME | 40 (44.4) | 50 (55.6) | | 62.80 ± 8.97 |  |  | | | 115 LR |  |  |  |  |  |  |  |  |  | |
|  | TaTME | 42 (46.7) | 48 (53.3) | | 63.78 ± 8.95 |  |  | | | 105 LR |  |  |  |  |  |  |  |  |  | |
| Miura et al. | L-TME | 8 (57.1) | 6 (42.9) | | 66 (34–77) | 21.4 (18.6–28.8) | 8 (57.1) | | | 5.7 (3.5–8.0) |  |  |  |  |  |  |  |  |  | |
|  | R-TME | 28 (80.0) | 7 (20.0) | | 65 (37–75) | 23.0 (18.7–31.4) | 21 (60.0) | | | 5.5 (2.5–8.0) |  |  |  |  |  |  |  |  |  | |
|  | TaTME | 14 (93.3) | 1 (6.7) | | 70 (38–81) | 23.3 (19.1–28.9) | 7 (46.7) | | | 5.0 (3.0–6.0) |  |  |  |  |  |  |  |  |  | |
| Ng et al. | O-TME | 15 (60.0) | 10 (40.0) | | 66.7 ± 12.4 | 21.4 ± 3.5 |  | | | 3 LR |  |  |  |  |  |  |  |  |  | |
|  | L-TME | 30 (61.2) | 19 (38.8) | | 65.6 ± 11.3 | 22.4 ± 3.4 |  | | | 8 LR |  |  |  |  |  |  |  |  |  | |
| Ozeki et al. | O-TME | 22 (100.0) | 0 (0.0) | | 62 ± 8.34 | 22 ± 4.59 |  | | | 15 LR |  |  |  |  |  | 21 (95.5) |  |  |  | |
|  | R-TME | 15 (100.0) | 0 (0.0) | | 58 ± 7.65 | 23 ± 1.75 |  | | | 10 LR |  |  |  |  |  | 15 (100.0) |  |  |  | |
| Park et al. | L-TME | 32 (100.0) | 0 (0.0) | |  | 23.6 (2.7) | 9 (28.1) | | |  |  |  |  |  |  |  |  |  |  | |
|  | R-TME | 32 (100.0) | 0 (0.0) | |  | 23.8 (2.3) | 15 (46.9) | | |  |  |  |  |  |  |  |  |  |  | |
| Rubinkiewicz et al. | L-TME | 22 (68.8) | 10 (31.2) | | 64 [58–67] | 26.5 [23.8–30.6] | 19 (59.4) | | | 4 [3–5]* |  |  |  |  |  | 2 (6.3) | 16 (50.0) | 5 (15.6) |  | |
|  | TaTME | 22 (68.8) | 10 (31.2) | | 60 [51–67] | 26 [22.8–29.7] | 18 (56.3) | | | 3 [2–4]* |  |  |  |  |  | 3 (9.4) | 15 (46.9) | 6 (18.8) |  | |
| Tang et al. | L-TME | 39 (100.0) | 0 (0.0) | | 46.01 ± 9.37 | 22.03 ± 2.51 | 11 (28.2) | | | 5.82 ± 2.43 |  |  |  |  |  | 27 (69.2) | 12 (30.8) |  |  | |
|  | R-TME | 38 (100.0) | 0 (0.0) | | 47.75 ± 9.62 | 21.34 ± 2.67 | 9 (23.7) | | | 6.33 ± 2.21 |  |  |  |  |  | 22 (57.9) | 16 (42.1) |  |  | |
| *Abbreviations: ASA,* American Society of Anesthesiology; *BMI,* body mass index; *L-TME,* laparoscopic total mesorectal excision; *LR,* low rectums; *n,* number of patients; *O-TME,* open total mesorectal excision; *R-TME,* robotic total mesorectal excision; *TaTME,* transanal total mesorectal excision; *-,* not available. * Median, IQR. | | | | | | | | | | | | | | | | | | | |  |

| **Supplementary Table 2:** Operative data and postoperative data | | | | | | | | | | | | | | | | | | | |
| --- | --- | --- | --- | --- | --- | --- | --- | --- | --- | --- | --- | --- | --- | --- | --- | --- | --- | --- | --- |
|  |  | Procedure, n (%) | | |  | |  | | | |  |  | Clavien-Dindo Classification, n (%) | | | | | | |
| First author | Technique | LAR | APR | Other^a^ | Operating time, mean (min) | Stomy formation, n (%) | | Conversions, n (%) | CRM+, n (%) | Number of surgeons | Surgeon’s experience | Complication rate, n (%) | | 0 | I | II | III | IV | V |
| D’Annibale et al. | L-TME | 50 (100.0) | 0 (0.0) | 0 (0.0) | 280 (240–350) |  | | 6 (12.0) | 6 (12.0) | 1 | Well trained. | 11 (22.0) | |  |  |  |  |  |  |
|  | R-TME | 50 (100.0) | 0 (0.0) | 0 (0.0) | 270 (240–315) |  | | 0 (0.0) | 0 (0.0) |  |  | 5 (10.0) | |  |  |  |  |  |  |
| Feng et al. | L-TME | 0 (0.0) | 173 (100.0) | 0 (0.0) | 195 (160−238) | 173 (100.0) | | 5 (2.9) | 0 (0.0) | Multiple. | >50 | 41 (23.7) | |  |  | 28 (16.2) | 12 (6.9) |  |  |
|  | R-TME | 0 (0.0) | 174 (100.0) | 0 (0.0) | 205 (195−220) | 174 (100.0) | | 0 (0.0) | 0 (0.0) |  | >50 | 23 (13.2) | |  |  | 16 (9.2) | 7 (4.0) |  |  |
| Galata et al. | L-TME | 28 (84.8) | 0 (0.0) | 5 (15.2) | 324 ± 80.9 (193–491) | 31 | | 0 (0.0) | 0 (0.0) | 3 | Considerable. | 12 (36.4) | | 21 (63.6) | 0 (0.0) | 5 (15.2) | 7 (21.2) | 0 (0.0) | 0 (0.0) |
|  | R-TME | 14 (77.8) | 0 (0.0) | 4 (22.2) | 394 ± 78.5 (252–535) | 15 | | 4 (22.2) | 1 (5.6) |  | Limited. | 6 (33.3) | | 12 (66.7) | 0 (0.0) | 3 (16.7) | 3 (16.7) | 0 (0.0) | 0 (0.0) |
| Grass et al. | R-TME | 48 (87.3) | 7 (12.7) | 0 (0.0) | 247.0 ± 88.0 | 39 (70.8) | | Excl. | 1 | NA | >40 | 13 (23.6) | |  |  |  |  |  |  |
|  | TaTME | 65 (100.0) | 0 (0.0) | 0 (0.0) | 297.8 ± 85.0 | 65 (100) | | Excl. | 0 | NA | >40 | 21 (32.3) | |  |  |  |  |  |  |
| Heijden et al. | L-TME | 55 (100.0) | 0 (0.0) | 0 (0.0) | NA | NA | | 5 (9.1) | NA | NA | NA | NA | |  |  |  |  |  |  |
|  | TaTME | 55 (100.0) | 0 (0.0) | 0 (0.0) | NA | NA | | 2 (3.6) | NA | NA | NA | NA | |  |  |  |  |  |  |
| Hur et al. | O-TME | 19 (86.4) | 0 (0.0) | 3 (13.6) | 155 ± 22.7 | 6 (14.6) | | 0 (0.0) |  | 1 | Experience. | 5 (12.2) | |  |  |  |  |  |  |
|  | L-TME | 26 (92.9) | 0 (0.0) | 2 (7.1) | 175 ± 31.1 | 8 (14.3) | | 0 (0.0) |  |  | Experience. | 6 (10.7) | |  |  |  |  |  |  |
| Jayne et al. | L-TME | 165 (71.7) | 45 (19.6) | 19 (8.3) | 261.0 (83.24) | 206 | | 28/230 (12.2) | 14/224 (6.3) | 40 | >10 | 73/230 (31.7) | |  |  |  |  |  |  |
|  | R-TME | 152 (64.4) | 52 (22.0) | 28 (11.9) | 298.5 (88.71) | 195 | | 19/236 (8.1) | 12/235 (5.1) |  | >10 | 78/236 (33.1) | |  |  |  |  |  |  |
| Kim et al. (AoS) | L-TME | 70 (75.3) | 22 (23.6) | 1 (1.1) | 227.8 (65.6) | 70 (95.9) | | 0 (0.0) | 4 (5.5) | 3 | >500 | 17 (23.3) | |  | 3 (4.1) | 10 (13.7) | 4 (5.5) |  |  |
|  | R-TME | 65 (72.2) | 25 (27.8) | 0 (0.0) | 339.2 (80.1) | 65 (98.5) | | 1 (1.5) | 4 (6.1) |  | >30 | 23 (34.8) | |  | 6 (9.1) | 11 (16.7) | 6 (9.1) |  |  |
| Kim et al. (ASO) | L-TME | 38 (97.4) | 0 (0.0) | 1 (2.6) | NA | NA | | NA | NA | 1 | Experience. | NA | |  |  |  |  |  |  |
|  | R-TME | 29 (96.7) | 0 (0.0) | 1 (3.3) | NA | NA | | NA | NA |  | Experience. | NA | |  |  |  |  |  |  |
| Kim et al. (CRD) | L-TME | 91 (70.0) | 2 (1.5) | 37 (28.5) | NA | 37 (28.5) | | NA | 2 (1.5) | NA | NA | 26 (20.0) | |  |  |  |  |  |  |
|  | R-TME | 90 (69.2) | 2 (1.5) | 38 (29.2) | NA | 43 (33.1) | | NA | 2 (1.5) | NA | NA | 23 (17.7) | |  |  |  |  |  |  |
| Liu et al. | L-TME | 36 (80) | 6 (13.3) | 3 (6.7) | 205.5 ± 85.0 | 35 (77.8) | | 3 (6.8) | 4 (8.8) | 1 | 200 | NA | |  |  |  |  |  |  |
|  | Microhand | 42 (97.7) | 1 (2.3) | 0 (0.0) | 235.0 ± 70.5 | 33 (76.7) | | 1 (2.3) | 3 (6.9) |  | 35 | NA | |  |  |  |  |  |  |
|  | Da Vinci | 45 (95.7) | 2 (4.3) | 0 (0.0) | 230.1 ± 75.5 | 37 (78.7) | | 1 (2.2) | 3 (6.4) |  | 30 | NA | |  |  |  |  |  |  |
| Macháčková et al. | L-TME | 39 (100.0) | Excl. | 0 (0.0) | 231.8 ± 64.3 | 39 (100.0) | | Excl. | NA | Multiple. | Well trained. | 4 (10.3) | |  |  |  |  |  |  |
|  | R-TME | 27 (100.0) | Excl. | 0 (0.0) | 279.2 ± 58.1 | 27 (100.0) | | Excl. | NA |  | Well trained. | 4 (14.8) | |  |  |  |  |  |  |
| Mei et al. | L-TME | 0 (0.0) | 90 (100.0) | 0 (0.0) | NA | NA | | NA | NA | 1 | NA | NA | |  |  |  |  |  |  |
|  | TaTME | 90 (100.0) | 0 (0.0) | 0 (0.0) | NA | NA | | NA | NA |  | NA | NA | |  |  |  |  |  |  |
| Miura et al. | L-TME | 14 (100.0) | 0 (0.0) | 0 (0.0) | 430 (185–639) | NA | | NA | NA | 1 | NA | NA | |  |  |  |  |  |  |
|  | R-TME | 35 (100.0) | 0 (0.0) | 0 (0.0) | 465 (299–631) | NA | | NA | NA | 2 | NA | NA | |  |  |  |  |  |  |
|  | TaTME | 15 (100.0) | 0 (0.0) | 0 (0.0) | 317 (170–396) | NA | | NA | NA | 1 | NA | NA | |  |  |  |  |  |  |
| Ng et al. | O-TME | 12 (48.0) | 0 (0.0) | 13 (52.0) | NA | 13 (52.0) | | Excl. | NA | NA | Experienced. | 13 (52) | |  |  |  |  |  |  |
|  | L-TME | 24 (49.0) | 0 (0.0) | 25 (51.0) | NA | 25 (51.0) | | Excl. | NA | NA | Experienced. | 17 (34.7) | |  |  |  |  |  |  |
| Ozeki et al. | O-TME | 16 (72.7) | 3 (13.6) | 3 (13.6) | 312 (166–548) | 17 | | NA | NA | NA | NA | 14 (63.6) | |  |  |  |  |  |  |
|  | R-TME | 12 (80.0) | 0 (0.0) | 3 (20.0) | 459 (326–833) | 9 | | NA | NA | 2 | NA | 7 (46.7) | |  |  |  |  |  |  |
| Park et al. | L-TME | 23 (71.9) | 1 (3.1) | 8 (25.0) | NA | 3 (9.4) | | NA | NA | 1 | >500 | NA | |  |  |  |  |  |  |
|  | R-TME | 22 (68.8) | 1 (3.1) | 9 (28.1) | NA | 3 (9.4) | | NA | NA |  | NA | NA | |  |  |  |  |  |  |
| Rubinkiewicz et al. | L-TME | 23 (100.0) | Excl. | 0 (0.0) | 212 (IQR: 180–250) | 23 (100.0) | | NA | 0 (0.0) | 1 | Expert skills. | 6 (26.1) | |  | 0 (0.0) | 2 (8.7) | 4 (17.4) | 0 (0.0) | 0 (0.0) |
|  | TaTME | 23 (100.0) | Excl. | 0 (0.0) | 252 (IQR: 190–300) | 23 (100.0) | | NA | 1 (4.5) |  | Cadaver courses. | 5 (21.7) | |  | 0 (0.0) | 1 (4.3) | 2 (8.7) | 1 (4.3) | 0 (0.0) |
| Tang et al. | L-TME | 31 (79.5) | 8 (20.5) | 0 (0.0) | NA | 7 (17.9) | | Excl. | NA | 1 | NA | NA | |  |  |  |  |  |  |
|  | R-TME | 32 (84.2) | 6 (15.8) | 0 (0.0) | NA | 8 (21.1) | | Excl. | NA |  | NA | NA | |  |  |  |  |  |  |
| *Abbreviations: APR,* abdominoperineal resection; *CRM+,* positive circumferential resection margin; *Excl,* excluded; L*-TME,* laparoscopic total mesorectal excision; *LAR,* low anterior resection; *n,* number of patients; *NA,* not applicable; *O-TME,* open total mesorectal excision; *R-TME,* robotic total mesorectal excision; *TaTME,* transanal total mesorectal excision; *-,* not available  * Data shows median values  ^a^ Other procedures include coloanal anastomosis, intersphincteric resection and Hartmann surgery  Stomy formation was only provided if reported in the article as percentage of the patients receiving restorative surgery. | | | | | | | | | | | | | | | | | | | |

| **Supplementary table 3:** Detailed overview of urinary function of the included studies | | | | | | | |
| --- | --- | --- | --- | --- | --- | --- | --- |
|  |  | IPSS |  |  |  |  |  |
| Study | Technique | Pre-op | 1m | 3m | 6m | 12m | 24m |
| Annibale et al. | L-TME | N=30 (male only), 3.50 (3) | N=30 (male only), 7.08 (3.5) |  |  | N=30 (male only), 4.2 (2.3) |  |
|  | R-TME | N=30 (male only), 3.24 (2.7) | N=30 (male only), 6.71 (5.9) |  |  | N=30 (male only), 3.53 (2.5) |  |
|  |  |  | NS |  |  | NS (0.886) |  |
| Feng et al. | L-TME | N=169, 0 (0-7.5) | N=169, 11 (7-18) | N=165, 7 (1-12) | N=162, 5 (0.8-8.3) |  |  |
|  | R-TME | N=172, 0 (0-7) | N=170, 8 (3.8-14) | N=166, 4 (0-10) | N=162, 2 (0-9) |  |  |
|  |  |  | RS (0.001) | RS (0.004) | RS (0.031) |  |  |
| Galata et al. | L-TME |  |  |  |  | ∆ N=21, − 1.3 ± 5.57 (− 9–16) |  |
|  | R-TME |  |  |  |  | ∆ N=11, 0.7 ± 4.9 (− 12–7) |  |
|  |  |  |  |  |  | NS (0.334) |  |
| Grass et al. | R-TME | ♂: 4.0 +/- 4.6 \|  ♀: 2.9 +/- 2.3 |  |  |  | ♂: 6.2 +/- 3.9. ∆ 5.1±0.9.\|  ♀: 3.8 +/- 2.9. ∆ 0.1±2.2. |  |
|  | TaTME | ♂: 0.5 +/- 1.1 \|  ♀: 0.3 +/- 0.6 |  |  |  | ♂: 2.2 +/- 2.8. ∆ 0.3±1.0.\|  ♀: 2.3 +/-2.9. ∆ 0.0±1.9. |  |
|  |  |  |  |  |  | ∆ ♂ TaS (<0.001). |  |
|  |  |  |  |  |  | ∆ ♀ NS (0.961). |  |
| Hur et al. | O-TME | N=41, 9.3 +/- 4.6 | N=41, 14.0 +/- 8.2 |  | N=41, 9.9 +/- 4.5 | N=41, 9.6 +/- 5.3 |  |
|  | L-TME | N=56, 8.3 +/- 5.3 | N=56, 12.3 +/- 5.2 |  | N=56, 9.2 +/- 5.6 | N=56, 8.4 +/- 4.6 |  |
|  |  |  | NR |  | NR |  |  |
| Jayne et al.* | L-TME |  |  |  | 0.743* |  |  |
|  | R-TME |  |  |  |  |  |  |
|  |  |  |  |  | NS (0.27) |  |  |
| Kim et al. (ASO) | L-TME | N=39, 7.95 +/- 5.61 | N=39, 12.79 +/- 7.2 | NR | N=39, 8.2 +/- 6.3 | NR |  |
|  | R-TME | N=30, 6.43 +/- 5.27 | N=30, 11.40 +/- 6.62 | N=30, 8.36 +/- 5.5 | NR | NR |  |
|  |  |  | NS | RS (0.036) | NS | NS |  |
| Kim et al. (CRD) | L-TME | N=130, 4.4 (3.8) |  | N=130, 9.0 (7.3) | N=130, 7.9 (7.3) | N=130, 5.7 (6.7) |  |
|  | R-TME | N=130, 4.4 (4.8) |  | N=130, 8.1 (6.8) | N=130, 6.3 (4.6) | N=130, 5.0 (5.6) |  |
|  |  |  |  | NS | RS (0.019) | NS |  |
| Liu et al. | L-TME | 6.3 ± 0.9 | 14.3 ± 1.5 |  |  |  |  |
|  | Microhand | 6.5 ± 1.0 | 13.3 ± 1.6 | 6.5 ± 1.0 |  |  |  |
|  | Da Vinci | 6.5 ± 2.0 | 13.6 ± 1.7 | 6.5 ± 2.0 |  |  |  |
|  |  |  | NR | RS (0.001)^3^ |  |  |  |
|  |  |  |  | RS (0.001)^4^ |  |  |  |
| Macháčková et al. | L-TME | N=24 (only males), 3.8 ±3.6 |  |  | N=24 (only males), 6.1 ±6.3 | N=24 (only males), 5.8 ±6.0 | N=24 (only males), 5.1 ±5.6 |
|  | R-TME | N=17 (only males), 5.8 ±4.0 |  | . | N=17 (only males), 8.6 ±5.3 | N=17 (only males), 7.7 ±4.9 | N=17 (only males), 7.5 ±4.9 |
|  |  |  |  |  | NS (0.090) | NS (0.125) | NS (0.085) |
| Miura et al. | L-TME | N=14, 5.5 (1-21) |  |  | N=11, 8 (0–29) |  |  |
|  | R-TME | N=35, 5 (0-27) |  |  | N=33, 3 (0–23) |  |  |
|  | TaTME | N=15, 7 (3-29) |  |  | N=8, 3 (1–16) |  |  |
|  |  |  |  |  | NS (0.09)^1^ |  |  |
|  |  |  |  |  | NS (0.35)^2^ |  |  |
| Ozeki et al.** | O-TME | N=22 (only males), 6 (2–14)** |  | N=22 (only males), 8.5 (4.7–20)** | N=22 (only males), 5.5 (1.7–12)** | N=22 (only males), 3.5 (0–9.7)** |  |
|  | R-TME | N=15 (only males), 6 (2–11)** |  | N=15 (only males), 7 (4–10)** | N=15 (only males), 4 (1–10)** | N=15 (only males), 6 (2–9)** |  |
|  |  |  |  |  |  |  |  |
| Park et al. | L-TME | N=32 (only males), 8.2 +/- 2.7 |  | N=32 (only males), 11.4 +/- 4.3 | N=32 (only males), 10.5 +/- 4.3 | N=32 (only males), 10.4 +/- 4.5 |  |
|  | R-TME | N=32 (only males), 8.5 +/- 3.0 |  | N=32 (only males), 10.7 +/- 3.7 | N=32 (only males), 9.8 +/- 3.3 | N=32 (only males), 9.5 +/- 3.4 |  |
|  |  |  |  | NS (0.458) | NS (0.493) | NS (0.399) |  |
| Tang et al. | L-TME | N=39 (only males), 7.04 ± 2.48 |  | N=39 (only males), 12.21 ± 2.62 | N=39 (only males), 9.95 ± 3.01 | N=39 (only males), 9.12 ± 2.64 |  |
|  | R-TME | N=38 (only males), 7.12 ± 3.05 |  | N=38 (only males), 11.65 ± 2.93 | N=38 (only males), 7.82 ± 2.25 | N=38 (only males), 7.62 ± 2.5 |  |
|  |  |  |  | NS (0.379) | RS (0.006) | RS (0.012) |  |
| *Abbreviations:*  *IPSS,* International Prostate Symptom Score; *L-TME,* laparoscopic total mesorectal excision; *m*, months; *N*, number of patients; *NR*, not reported; *NS*, non-significant; *O-TME,* open total mesorectal excision; *Post-op*, postoperative; *Pre-op*, preoperative; *R-*TME, robotic total mesorectal excision; *RS*, robot significantly better; *TaTME,* transanal total mesorectal excision; *TaS*, transanal significantly better. *** calculated L-TME minus R-TME; **** provided median change in IPSS; ♂, male; ♀, female; ^1^L-TME vs. R-TME; ^2^R-TME vs. TaTME; ^3^Microhand vs. L-TME; ^4^da Vinci vs. L-TME; ∆*,* delta. | | | | | | | |

| **Supplementary table 4:** Detailed overview of sexual function of the included studies | | | | | | | | | | | | | |
| --- | --- | --- | --- | --- | --- | --- | --- | --- | --- | --- | --- | --- | --- |
|  |  | IIEF |  |  |  |  |  | FSFI |  |  |  |  |  |
| Study | Technique | Pre-op | 1m | 3m | 6m | 12m | 24m | Pre-op | 3m | 6m | 12m | 24m |  |
| Feng et al. | L-TME | N=47, 21 (16-23) |  | N=44, 5 (4-8) | N=43, 11 (6-13) | N=41, 15 (8.5-20) |  | N=26, 21.2 (17.0-27.0) | N=23, 8.7 (2.0-12.0) | N=22, 12.0 (9.5-14.5) | N=21, 15.3 (10.5-17.8) |  |  |
|  | R-TME | N=56, 21 (15-23) |  | N=54, 10 (5-11) | N=52, 14 (10-15) | N=49, 18 (13.5-21) |  | N=26, 22.2 (18.0-26.4) | N=24, 12.8 (8.2-14.4) | N=23, 16.0 (14.0-17.6) | N=23, 19.3 (16.8-21.6) |  |  |
|  |  |  |  | RS (<0.001) | RS (0.005) | RS (0.014) |  |  | RS (0.001) | RS (0.002) | RS (0.003) |  |  |
| Galata et al. | L-TME |  |  |  |  | ∆ N=21, − 4.6 ± 29.9  (− 39–53) |  |  |  |  | ∆ N=21, − 8.3 ± 10.0  (− 19–1.5) |  |  |
|  | R-TME |  |  |  |  | ∆ N=11, − 13 ± 18.9  (− 29–8) |  |  |  |  | ∆ N=11, 2.3 ± 6.6  (− 5.9–13.3) |  |  |
|  |  |  |  |  |  | ∆ NS (0.682) |  |  |  |  | ∆ NS (0.079) |  |  |
| Grass et al. | R-TME | 26.0 +/- 17.8 |  |  |  | 19.2 +/- 18.4 |  | 20.8 +/- 11.8 |  |  | 15.7 +/- 11.9.  ∆ 5.2±4.6. |  |  |
|  | TaTME | 36.1 +/- 28.6 |  |  |  | 34.8 +/- 27.9 |  | 9.1 +/- 8.8 |  |  | 8.5 +/- 8.5.  ∆ 10.5±6.4. |  |  |
|  |  |  |  |  |  | NR |  |  |  |  | ∆ NS (0.254). |  |  |
| Hur et al. | O-TME | N=22, 55.2 +/- 9.8 | N=22, 20.9 +/- 13.7 |  | N=22, 37.8 +/- 14.7 | N=22, 48.7 +/- 15.9 |  |  |  |  |  |  |  |
|  | L-TME | N=28, 55.4 +/- 9.0 | N=28, 21.7 +/- 11.2 |  | N=28, 48.7 +/- 12.9 | N=28, 52.2 +/- 11.7 |  |  |  |  |  |  |  |
|  |  | NR | NR |  | NR | NR |  |  |  |  |  |  |  |
| Jayne et al.* | L-TME |  |  |  | 0.802* |  |  |  |  | 1.231* |  |  |  |
|  | R-TME |  |  |  | NS (0.75) |  |  |  |  | NS (0.60) |  |  |  |
|  |  |  |  |  | NS (0.75)* |  |  |  |  | NS (0.60)* |  |  |  |
| Kim et al. (ASO) | L-TME | N=20, 51.55 ± 11.98 | N=20, 16.35 +/- 15.7 |  |  | N=20, 46.00 +/- 16.9 |  |  |  |  |  |  |  |
|  | R-TME | N=18, 54.62 +/- 14.2 | N=18, 19.89 +/- 18.8 |  | N=18, 44.61 +/- 13.76 |  |  |  |  |  |  |  |  |
|  |  |  | NS | NS | NS | NS |  |  |  |  |  |  |  |
| Kim et al.  (CRD) | L-TME | N=48, 17.2 (4.7) |  | N=48, 12.6 (5.6) | N=48, 14.1 (5.2) | N=48, 15.6 (4.7) |  |  |  |  |  |  |  |
|  | R-TME | N=48, 18.4 (4.7) |  | N=48, 13.4 (6.4) | N=48, 15.7 (6.2) | N=48, 16.3 (6.0) |  |  |  |  |  |  |  |
|  |  |  |  | NS | NS | NS |  |  |  |  |  |  |  |
| Liu et al. | L-TME | 50.6 ± 13.3 | 24.5 ± 12.1 |  | 29.3 ± 4.3 |  |  | 28.8 ± 2.0 |  | 26.0 ± 6.4 |  |  |  |
|  | Microhand | 52.7 ± 13.3 | 26.3 ± 12.1 |  | 49.8 ± 11.0 |  |  | 28.4 ± 1.7 |  | 28.7 ± 1.5 |  |  |  |
|  | Da Vinci | 53.2 ± 13.0 | 27.6 ± 12.0 |  | 52.1 ± 11.0 |  |  | 28.2 ± 1.6 |  | 27.8 ± 1.5 |  |  |  |
|  |  |  |  |  | RS (0.001)^1^ |  |  |  |  | NS (0.833)^1^ |  |  |  |
|  |  |  |  |  | RS (0.001)^2^ |  |  |  |  | NS (0.683)^2^ |  |  |  |
| Macháčková  et al. | L-TME | N=15, 23.5 ±0.9 |  |  | N=15, 21.9 ±3.1 | N=15, 21.7 ±3.2 | N=15, 22.9 ±2.6 | N=11, 31.1 ±2.9 |  | N=11, 28.2 ±5.4 | N=11, 28.9 ±5.0 | N=11, 30.8 ±6.0 |  |
|  | R-TME | N=11, 22.9 ±2.2 |  |  | N=11, 20.5 ±4.0 | N=11, 21.2 ±3.1 | N=11, 21.4 ±3.4 | N=6, 32.1 ±3.4 |  | N=6, 31.9 ±3.5 | N=6, 32.1 ±3.2 | N=6, 32.9 ±2.9 |  |
|  |  |  |  |  | NS (0.45) | NS (0.614) | NS (0.302) |  |  | NS (0.175) | NS (0.175) | NS (0.33) |  |
| Ozeki et al.** | O-TME | N=22, 21 (10–60)** |  | N=22, 12 (8.5–32)** | N=22, 16 (9–32)** | N=22, 12 (8.7–32)** |  |  |  |  |  |  |  |
|  | R-TME | N=15, 34 (11–60)** |  | N=15, 32 (10–39)** | N=15, 24 (13–35)** | N=15, 27 (14–38)** |  |  |  |  |  |  |  |
|  |  |  |  | NR | NR | NR |  |  |  |  |  |  |  |
| Park et al. | L-TME | N=20, 18.6 +/- 5.0 |  | N=20, 9.1 +/- 6.5 | N=20, 9.4 +/- 6.6 | N=20, 13.7 +/- 7.9 |  |  |  |  |  |  |  |
|  | R-TME | N=20, 18.9 +/- 4.3 |  | N=20, 11.5 +/- 7.6 | N=20, 14.1 +/- 6.1 | N=20, 15.1 +/- 5.1 |  |  |  |  |  |  |  |
|  |  |  |  | NS (0.301) | RS (0.024) | NS (0.510) |  |  |  |  |  |  |  |
| Tang et al. | L-TME | N=39, 22.93 ± 3.82 |  | N=39, 13.92 ± 3.62 | N=39, 16.75 ± 3.26 | N=39, 19.95 ± 3.03 |  |  |  |  |  |  |  |
|  | R-TME | N=38, 22.23 ± 3.65 |  | N=38, 14.87 ± 3.27 | N=38, 18.55 ± 3.45 | N=38, 21.22 ± 3.06 |  |  |  |  |  |  |  |
|  |  |  |  | NS (0.231) | RS (0.021) | NS (0.071) |  |  |  |  |  |  |  |
| *Abbreviations:* *FSFI,* female sexual function index; *IIEF,* International Index of Erectile Function; *L-TME,* laparoscopic total mesorectal excision; *m*, months; *N*, number of patients; *NR*, not reported; *NS*, non-significant; *O-TME,* open total mesorectal excision; *Pre-op*, preoperative; *R-*TME, robotic total mesorectal excision; *RS*, robot significantly better; *TaTME,* transanal total mesorectal excision. *** calculated L-TME minus R-TME; **** provided median change in IIEF; ^1^Microhand vs. L-TME; ^2^da Vinci vs. L-TME; ∆*,* delta. | | | | | | | | | | | | | |

| **Supplementary Table 5:** Detailed overview of fecal function of the included studies | | | | | | | | |  |
| --- | --- | --- | --- | --- | --- | --- | --- | --- | --- |
|  |  | LARS | | | Wexner | | | |  |
| Study | Technique | Pre-op | 6m | 12m | | Pre-op | 6m | 12m | |
| Grass et al. | R-TME | 8.9 +/- 11.7 |  | 3.8 +/- 1.9. ∆ 4.3±2.2 | | 2.8 +/- 3.2 |  | 2.0 +/- 1.2. ∆ 1.0±0.7 | |
|  | TaTME | 1.9 +/- 5.9 |  | 13.4 +/- 12.4. ∆ 9.8±1.5 | | 0.3 +/- 0.9 |  | 3.8 +/- 4.4. ∆ 2.7±0.5 | |
|  |  |  |  | ∆ RS (0.038) | |  |  | ∆ NS (0.095) | |
| Heijden et al. | L-TME | N=55, 18.2 (12.9) |  | N=55, 25.4 (11.6) | |  |  |  | |
|  | TaTME | N=55, 23.4 (11.2) |  | N=55, 30.6 (9.5) | |  |  |  | |
|  |  |  |  | LS (0.010) | |  |  |  | |
| Miura et al. | L-TME | N=14, 11 (0-41) | N=11, 35 (31-41) |  | | N=14, 1 (0-18) | N=11, 11 (6-19) |  | |
|  | R-TME | N=35, 11 (0-39) | N=33, 35 (11-41) |  | | N=35, 1 (0-16) | N=33, 10 (0-20) |  | |
|  | TaTME | N=15, 15 (0-38) | N=8, 38 (33-42) |  | | N=15, 2 (0-4) | N=8, 12 (4-17) |  | |
|  |  |  | NS (0.08)^1^ |  | |  | NS (0.16)^1^ |  | |
|  |  |  | NS (0.22)^2^ |  | |  | NS (0.13)^2^ |  | |
| Rubinkiewicz et al.* | L-TME | N=23, 0 [0–5]* | N=23, 30 [21–34]* |  | | N=23, 0 [0–1]* | N=23, 7 [3–11]* |  | |
|  | TaTME | N=23, 5 [0–21]* | N=23, 29 [24–34]* |  | | N=23, 0 [0–2]* | N=23, 8 [4–12]* |  | |
|  |  |  | NS (0.76)* |  | |  | NS (0.83)* |  | |
| *Abbreviations:* *L-TME,* laparoscopic total mesorectal excision; *LARS,* Low Anterior Resection Syndrome; *LS,* laparoscopic significantly better; *N*, number of patients; *NS*, non-significant; *Post-op*, postoperative; *Pre-op*, preoperative; *R-TME*, robotic total mesorectal excision; *RS*, robot significantly better; *TaTME,* transanal total mesorectal excision. *** provided median LARS/Wexner scores; ∆*,* delta; ^1^L-TME vs. R-TME; ^2^R-TME vs. TaTME. | | | | | | | | |  |

| **Supplementary Table 6:** Detailed overview of quality of life of the included studies | | | | | | |
| --- | --- | --- | --- | --- | --- | --- |
|  |  | Global Health Score | | | | |
| Study | Technique | Pre-op | 4m | 6m | 8m | 12m |
| Heijden et al. | L-TME |  |  |  |  | N=55, 78.5 (16.9) |
|  | TaTME |  |  |  |  | N=55, 78.2 (18.2) |
|  |  |  |  |  |  | NS |
| Kim et al. (AoS) | L-TME |  |  |  |  | NR |
|  | R-TME |  |  |  |  | NR |
|  |  |  |  |  |  | NS |
| Kim et al. (CRD) | L-TME |  |  |  |  | NR |
|  | R-TME |  |  |  |  | NR |
|  |  |  |  |  |  | NS |
| Mei et al. | L-TME | N=90, 58.70 ± 10.22 |  | N=90, 32.04 ± 9.51 |  | N=90, 30.09 ± 8.80 |
|  | TaTME | N=90, 60.92 ± 9.15 |  | N=90, 35.86 ± 25.85 |  | N=90, 54.62 ± 10.00 |
|  |  |  |  | NS (0.189) |  | TaS (<0.001) |
| Ng et al. | O-TME | N=25, 68.3 (5.2) | N=25, 47.3 (5.9) |  | N=25, 52.0 (5.9) | N=25, 61.0 (7.0) |
|  | L-TME | N=49, 72.4 (3.4) | N=49, 65.8 (3.6) |  | N=49, 73.6 (3.8) | N=49, 71.1 (3.4) |
|  |  |  | LS (0.009) |  | LS (0.003) | NS (0.371) |
| *Abbreviations:* *L-TME,* laparoscopic total mesorectal excision; *LS*, laparoscopic significantly better; *m*, months; *N*, number of patients; *NR*, not reported; *NS*, non-significant; *O-TME,* open total mesorectal excision; *Post-op*, postoperative; *Pre-op*, preoperative; *R-*TME, robotic total mesorectal excision; *TaTME,* transanal total mesorectal excision; *TaS*, transanal significantly better. | | | | | | |
